# The connection between innervation and metabolic rearrangements in pancreatic cancer through serine

**DOI:** 10.3389/fonc.2022.992927

**Published:** 2022-12-13

**Authors:** Mengmeng Dong, Lidong Cao, Ranji Cui, Yingjun Xie

**Affiliations:** ^1^ Jilin Provincial Key Laboratory on Molecular and Chemical Genetics, The Second Hospital of Jilin University, Changchun, China; ^2^ Department of Hepatobiliary and Pancreatic Surgery, Second Hospital of Jilin University, Changchun, China; ^3^ Jilin Engineering Laboratory for Translational Medicine of Hepatobiliary and Pancreatic Diseases, Second Hospital of Jilin University, Changchun, China; ^4^ Department of Hepatobiliary and Pancreatic Surgery, Zhejiang Provincial Peoples Hospital, Hangzhou, China

**Keywords:** malignant tumors, pancreatic, innervation, metabolic rearrangements, cancer

## Abstract

Pancreatic cancer is a kind of aggressive tumor famous for its lethality and intractability, and pancreatic ductal adenocarcinoma is the most common type. Patients with pancreatic cancer often suffer a rapid loss of weight and abdominal neuropathic pain in their early stages and then go through cachexia in the advanced stage. These features of patients are considered to be related to metabolic reprogramming of pancreatic cancer and abundant nerve innervation responsible for the pain. With increasing literature certifying the relationship between nerves and pancreatic ductal adenocarcinoma (PDAC), more evidence point out that innervation’s role is not limited to neuropathic pain but explore its anti/pro-tumor functions in PDAC, especially the neural–metabolic crosstalks. This review aims to unite pancreatic cancer’s innervation and metabolic rearrangements with terminated published articles. Hopefully, this article could explore the pathogenesis of PDAC and further promote promising detecting or therapeutic measurements for PDAC according to the lavish innervation in PDAC.

## Background

Cancer is the most urgent public health problem worldwide, and it was estimated that 1,806,590 people will be diagnosed as cancer patients and 606,502 people will die of this deadly disease in America in 2020. Among these numbers, 57,600 cancer cases and 47,050 cancer deaths were attributed to pancreatic cancer in the United States (1). Moreover, pancreatic cancer has the lowest 5-year overall survival (OS) rate of about 9% to 10% of any other solid tumors. The percentage of incidence and mortality of pancreatic cancer is highest among people aged 65–74 for both sexes (2). The (age-adjusted) incidence and mortality rate of pancreatic cancer was 13.1 per 100,000 persons based on the database from 2013 to 2017 and 11.0 per 100,000 persons during 2014–2018. Statistically, men have a higher incidence than women (14.9 and 11.6 annually per 100,000 persons, respectively) as were the mortality rates (12.7 and 9.6 annually per 100,000 persons, respectively). Depending on the statistical analysis, the age-adjusted rates for new pancreatic cancer cases have been stable from 2008 to 2017; however, age-adjusted death rates have been increasing on average by 0.3% per year from 2009 to 2018 (2, 3). It is predicted that pancreatic cancer will be the second leading cause of cancer-related deaths by 2030 (4). Lately, it has exceeded breast cancer as the third leading cause of cancer death in all ages for both genders and the fourth leading cause of cancer death for males and females, respectively (1, 5).

**Figure 1:**
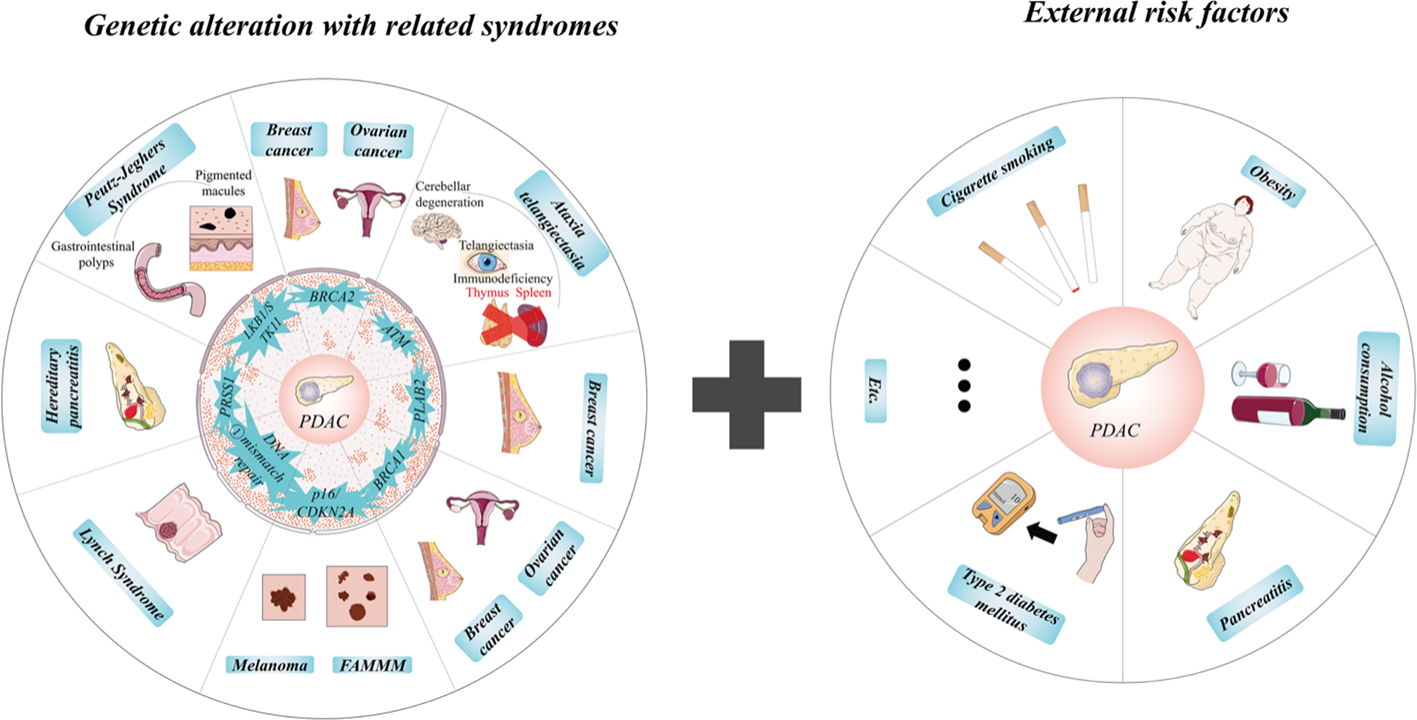
Internal and external risks of pancreatic ductal adenocarcinoma (PDAC). Notably, familial PDAC shows a remarkably rising risk of PDAC compared with the general population. Based on these altered genes and mutation-related syndromes, there is an excellent opportunity to quantify the risk of PDAC, which is essential for the clinical application of early detection and timely treatment of PDAC and screening for the risk of other malignant diseases. ①, DNA mismatch repair genes include hMSH2, hMLH1, hPMS1, hPMS2, and hMSH6/GTBP.

Pancreatic cancer, with the malignant neoplasm, has many histologic types, beginning with neoplasms originating from ductal and non-ductal cells. The former includes pancreatic ductal adenocarcinoma (PDAC), accounting for approximately 90% of total types and representing typical pancreatic cancer (6). Other PDAC-related carcinomas in neoplasm of ductal origin include adenosquamous carcinoma, osteoclastic giant cell carcinoma, colloid carcinoma, and medullary carcinoma. The last kind of original ductal neoplasms arises in tumoral intraepithelial neoplasms consisting of intraductal papillary mucinous neoplasm (IPMN), intraductal tubulopapillary neoplasm, and mucinous cystic neoplasm with associated invasive carcinoma. Intriguingly, a comment points out that 18% IPMNs and PDAC are found to be co-occurring in the same pancreas, which supports the concept of “field effect” (7), but neither genetically related nor representing a similar oncogenetic development (8). Acinar cell carcinoma, pancreatoblastoma, pancreatic neuroendocrine neoplasm, and solid pseudopapillary neoplasm are the other four kinds belonging to neoplasms of non-ductal origin (9, 10). Known as the most aggressive cancer, PDAC always shows enormous difficulties in diagnosis and therapy. The pancreas is located within the upper abdomen, so PDAC is extremely hard to be detected in its early stage by image measurements and resected by surgery because of the surrounding encased vessels. PDAC shows an aggressive biological nature of invasion and early metastasis. With rapid proliferation and growth limiting nutrition and energy intake, patients often suffer cachexia and pancreatic dysfunction for exocrine and endocrine (11). Moreover, PDAC has a desmoplastic stromal and flexible elasticity, and plasticity exhibits apparent resistance to chemotherapy and radiotherapy and has little target molecule (12, 13). Cigarette smoking, severe obesity (body mass index known as >35) (14), type 2 diabetes mellitus, alcohol consumption, and pancreatitis increase the risk of PDAC, and cigarette smoking is the most common one (15, 16). According to the environmental risk factors listed, genetic alterations play a more significant role in the appearance of PDAC. Among the PDAC patients with a pathogenic germline mutation, *BRCA2* (associated with breast and ovarian cancer) and *ATM* (related to ataxia–telangiectasia) are the top two genes (17, 18). Besides *PLAB2 *(correlated to breast cancer),* BRCA1 *(connected to ovarian and breast cancer), and *p16/CDKN2A* (associated with risk of melanoma and familial atypical multiple mole melanoma), DNA mismatch repair genes like *hMSH2*, *hMLH1*, *hPMS1*, *hPMS2*, and *hMSH6/GTBP* (Lynch syndrome), *PRSS1* (causing rare inherited hereditary pancreatitis), and *LKB1/STK11* (explaining about 80% of Peutz–Jeghers cases) also increased the estimated risk of PDAC (17, 19, 20). Notably, familial PDAC shows a remarkably rising risk of PDAC compared with the general population (21). Based on these altered genes and mutation-related syndromes, there is an excellent opportunity to quantify the risk of PDAC, which is essential for the clinical application of early detection and timely treatment of PDAC and screening for the risk of other malignant diseases (**Figure 1**).

PDAC presents few clinical symptoms before it evolves into an advanced stage; if any, those symptoms often lack specificity for one to make a diagnosis. Generally, these non-specific clinical symptoms include abdominal pain, jaundice, abnormal liver functions, diabetes, dyspepsia, nausea or vomiting, back pain, and weight loss (22). The most frequent feature of PDAC is abdominal pain related to neuropathic pain mainly caused by perineural invasion (PNI), defined as cancer cells diffusing at least 33% of the surrounding nerves, including the epineural, perineural, and endoneurial space of the nerve sheath (23). Invasion of PDAC cells impairs the neural sheath and reprograms neural density, causing neuropathic and inflammatory pain, whereby PNI suggests a metastatic route for PDAC metastasis and neuropathic pain. Interestingly, a study points out that PNI has the highest prevalence in PDAC within variant gastrointestinal malignancies and shows an impactful association with poor PDAC patient outcomes (24). The genetic analysis of PDAC also confirms frequent regulators of axon guidance (25), which is consistent with the correlation between neuronal supports and axonogenesis of PDAC.

PDAC has a desmoplastic and abundantly innervated tumor microenvironment (TME). However, the dense stromal leads to a nutrient-poor environment, rendering PDAC a plastic metabolic remodeling for oxygen and metabolites (26). The metabolic rearrangements of PDAC allow the tumor to survive in the specific environment and, more importantly, increase and overgrow in human bodies. PDAC cells would choose non-classic metabolic pathways (aerobic glycolysis, hexosamine biosynthetic pathways, *de novo* lipogenesis, *etc.*) for their energy and nutrient production under some circumstances (12). Accordingly, other cellular components such as pancreatic satellite cells (“reverse Warburg effect”—pancreatic satellite cells promoting metabolites for PDAC cells to produce energy) and neurons (known to release amino acid-derived neurotransmitters like serine *via* peripheral axons) also provide the metabolic needs for PDAC cells under nutrient-depleted conditions (27, 28).

## Nerve innervation in PDAC

**Figure 2:**
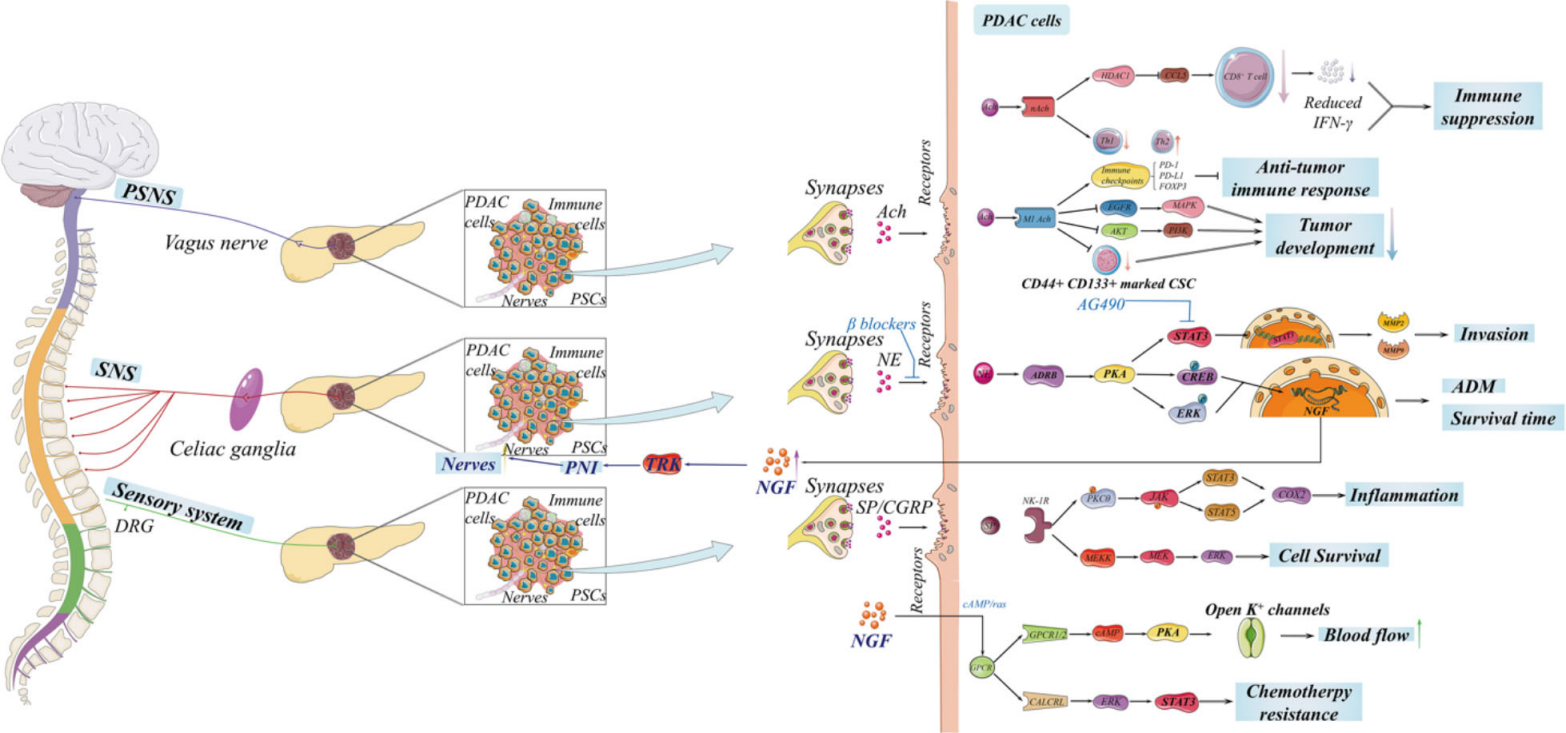
The innervation of the pancreas is complex, including the autonomic nervous system and sensory nerves, which is significant for tumorigenesis and the development of pancreatic ductal adenocarcinoma (PDAC). The sympathetic nervous and sensory systems promote the development of PDAC through neurotransmitters. Notably, neuropathic pain induced by PDAC activates sensory nerves in nerve impulses and then triggers transient receptor potential vanilloid 1 (TRPV1) to secrete substance P and calcitonin gene-related peptide. However, there is an extensive debate on the complicated role of parasympathetic nervous system in PDAC, and the entire signaling network remains to be discovered.

Organs like the pancreas are innervated by the autonomic nervous system consisting of two parts: the sympathetic nervous system (SNS) and the parasympathetic nervous system (PSNS) (29). Sensory nerves, compose of dorsal root ganglia (DRG), are responsible for receiving all nerve impulses from the pancreas. Sympathetic, sensory, and parasympathetic nerves disperse the whole pancreas and play individual roles (30, 31). Classically, the plexus leaving from the coeliac plexus and then entering the pancreas head innervates the head of the pancreas, and the plexus derived from the splenic plexus innervates the body and the tail of the pancreas (32). It has been proved that not only PDAC cells but also PanIN cells and PDAC patients demonstrate highly increased nerve penetration and neurotropism by KPC in *in vivo *murine and *in vitro *co-culture models (33–35). The abundant nerve innervation of the pancreas is the foundation of PNI, besides the precondition for interactions between nerves and PDAC cells (**Figure 2**).

### Sympathetic nerves

Sympathetic innervation in the pancreas comes from the sympathetic preganglionic neurons from the thoracic 5–10 segments of the spinal cord. They leave as sympathetic ganglia, become splanchnic nerves, and end in celiac ganglia (36). Sympathetic preganglionic neurons release acetylcholine (Ach) as functional neurotransmitters comprising cholinergic neurons. However, sympathetic postganglionic neurons releasing norepinephrine (NE) linking to adrenergic receptors on its downstream tissues as their functional neurotransmitters suggest that sympathetic postganglionic neurons should be classified as noradrenergic neurons (31, 37). It is widely accepted that catecholamines produced by sympathetic nerves as stress molecules could stimulate the proliferation of PDAC. An orthotopic mouse model, non-invasively tracked by *in vivo* optical imaging, is applied to evaluate the growth and proliferation of PDAC. This model greatly reappears the interlaced association of PDAC cells and microenvironment, and it indicates that pharmacological β-adrenergic is ample to reverse the activated β-adrenergic signaling caused by chronic stress responsible for the development of PDAC. After treating PDAC cells with isoproterenol, invasion-related genes like *matrix metalloprotease 2* (*MMP2*) and *MMP9* were assayed. These accelerating expressions collectively suggested that β-adrenergic signaling impacts the growth, progression, and invasion of PDAC *in vivo* (38). Another *in vitro *mouse model implanted a DRG nearby a colony of PDAC cells (including MIA PaCa-2 and BxPC-3 cell lines) in Matrigel, whose growth factors are depleted, and then applied with different concentrations of NE for 7 days (39). The model strongly shows that NE is critical in the PNI development of PDAC. Mechanistically, NE activates the STAT3 pathway through β-adrenergic receptors (ADRB) and its downstream PKA. After blocking the effect of STAT3 by phosphorylation inhibitor AG490, the expressions of STAT3 were attenuated, accompanied by the invasion-related protein levels of MMP9 and MMP2 and the secretion of neural growth factors (NGF) induced by NE. The *in vitro* experiments demonstrate that STAT3 blockade could inhibit NE-induced PDAC growth, migration, and PNI (39). It is well studied that sympathetic innervation impacts tumorigenesis and the growth of PDAC (mainly associated with NE produced by sympathetic postganglionic neurons) and breast cancers (associated with the expression of immune checkpoint molecules) (38–41). Additionally, a recent work aims to determine whether sympathetic neural signaling would affect the progression of PanIN (41). They utilized the genetically modified LSL-*Kras*+/LSL-G12D;*Pdx1*- [(KC) mice to simulate murine PanIN. After being treated by daily repeated immobilization, chronic restraint stress accelerated the systemic epinephrine levels and promoted PDAC carcinogenesis in the KC murine models. The common mediator of chronic stress is reported as β2-adrenergic receptor (ADRB2), which was also increasing in the pancreas of KC mice. Then, the LSL-*Kras*+/LSL-G12D;LSL-*Trp53*+/R172H;*Pdx1*-Cre (KPC) model, which could develop an advanced stage of PanIN, is established; these results claimed that ADRB2 blockade tremendously prolonged OS in KPC mice and that ADRB2 signaling is crucial for nerve growth in PDAC carcinogenesis. Mechanistically, adrenergic signaling related to catecholamine, specifically isoproterenol, induces PDAC development through acinar to ductal metaplasia (ADM), which is also described as the first step of PDAC carcinogenesis (42, 43), and promotes the secretion of neurotrophins (NT) through ADRB2 and the PKA/ERK pathways. Moreover, after assessing the expression of several NT, including NGF, brain-derived neurotrophic factor (BDNF), neurotrophic factor 3 (NTF3), NTF4, glial cell line-derived neurotrophic factor (GDNF), and Netrin-1 (encoded by *NTN1*), NGF demonstrated the highest expression in PDAC cell lines, KC mice, and KPC mice. Significantly, the blockade of the NGF/Trk pathway could inhibit proliferation, PNI, and invasion of PDAC, prolonging the OS for KPC mice (41, 44, 45). That hints us in utilizing β-blocker to suppress adrenergic signaling for highly innervated PDAC patients. Clinically speaking, treating postoperative PDAC patients of stages II and III with non-selective β-blocker reveals attenuated BDNF levels, decreased nerve density, and extended OS (41). In summary, sympathetic neuron-expressed NE promotes carcinogenesis and the development of PDAC while facilitating the secretion of NT, which conversely stimulates innervation in PDAC, followed by increased sympathetic related NE and PDAC growth. There is a feed-forward loop among sympathetic neurons, ADRB2 pathway, PDAC cells, and NT, and this loop could render PDAC sufficient neuroplasticity for innervation, proliferation, and growth. In that case, targeting this specific pathway gives us a novel strategy to treat the neoneurogenesis of PDAC. Coincidentally, this paper testified the possibility of targeting ADRB2 signaling to influence innervation in human PDAC to further improve the clinical outcomes of PDAC patients (41). Sahni and colleagues collected a cohort of PDAC patients who underwent surgical resection for their stage II and III tumors, and they were treated with non-selective β-blockers (NSBB), β-1 selective blocker (SB1B), and no β blockers (NBB). Then, the patients were retrospectively analyzed to compare their OS. Interestingly, patients with NSBB have no significant difference from those with SB1B in OS. However, they had a nearly double OS compared with patients with NBB. The immunohistochemistry analysis also confirms that there were fewer innervation (presented by neuronal marker S-100) (46) and decreased BDNF expressions in the NSBB group than in the NBB and SB1B groups. Notably, stages II and III PDAC patients who undergo surgical resection would benefit from NSBB and improve their clinical outcomes. Nevertheless, PDAC patients can barely benefit from β-blocker (NB) usage before diagnosis for their survival advantage. However, they acquire a clear survival advantage through constant β-blocker usage before and after diagnosis (*n* = 2,564) (47). Furthermore, another latest clinical research claims that NB usage is not correlated to accumulated PDAC risk but reduces the risk in long-term usage, especially NSBB usage (*n* = 4,113) (48). To date, these publications confirm the therapeutic effect of NSBB for the blockade of ADRB signaling associated with sympathetic relevant NE in PDAC and guarantee reliability and security in PDAC patients.

Although sympathetic preganglionic neurons are concluded into cholinergic neurons, sympathetic nerves have typically been considered to exert NE/ADRB signaling in PDAC. There is little literature to investigate sympathetic Ach in PDAC and other cancers to date. Ach, as a chemical messenger secreted from the ganglion synapse, directly binds to nicotinic acetylcholine receptors (nAchRs) at postganglionic neurons to induce the release of NE from postganglionic neurons. We believe that the cholinergic parts of sympathetic nerves have a little direct impact on the oncogenesis and neoneurogenesis of PDAC. Hypothetically, we deem that the secretion of sympathetic preganglionic Ach is straightly binding to nAchRs at postganglionic neurons, and superfluous Ach is sabotaged and devitalized by cholinesterase which barely leaks into the microenvironment nor has direct connections with cancer cells in PDAC or other cancers.

### Sensory nerves

Afferent sensory (afferent) nerves transmit sensory information from the pancreas to the central nervous system (CNS) and company with splanchnic nerves and vagi, whose neural bodies lie in DRG, the spinal afferents and nodose ganglia (NG), vagal afferents. Sensory nerves consist of unmyelinated fibers and have sensory and secretory functions (49, 50). It was observed that there is augmented axon density in PanIN lesions *in vivo*. Moreover, a microfluidic device was utilized to co-culture DRG neurons with PDAC cells and separate the axons (33). This co-cultured system allows axonal interactions and simulates intrapancreatic sensory innervation in human PDAC. Undoubtedly, accelerated sensory axons were recruited through PDAC cells, and an increased number of axons expressed synapsin proteins, suggesting functional axons with enhanced neurotransmitter release and transport. Intriguingly, the genetic and functional aberration of axons has a strong association with the outcome of PDAC patients and cancer progression in murine PDAC models (51). It was found that semaphorin exhibits an augmented expression, which is correlated to the poor OS of PDAC patients on univariate analysis. The same change occurred in early pancreatic tumorigenesis modeling by *in vivo* pancreatic injury and *in vitro* ADM, in contrast to normal pancreas (25). Taken together, utilizing antagonists against aberration of axon guidance genes and expression would significantly inhibit the initiation and progress of PDAC. DRG and NG contain sensory neurons sensitive to capsaicin, and capsaicin activates TRPV1 on the unmyelinated sensory fibers (52). Activated TRPV1 has a calcium preference, mainly leading to the release of substance P (SP) and calcitonin gene-related peptide (CGRP) (53). SP belongs to tachykinins encoded by *TAC-1*, also known as pre-pro-tachykinin-A (Ppt-a) (54). Neurokinin receptor 1 (NK-1R), encoded by *TACR1*, is one of the families of G-protein coupled receptors (GPCR)* *combined with SP, which then promotes several signaling pathways for the progress of cancer (55)—for instance, SP, along with its high-affinity target NK-1R, was augmented and activated HER2 in breast cancer cell lines (HER2+) and primary cell culture from breast cancer patients (HER2+). SP acted as a pro-inflammatory factor to modulate the expression of EGFR and HER2 for tumor malignancy. This transactivation is significantly reduced by the inhibition of NK-1R RNA expression, a chemical inhibitor of NK-1R, and inhibition of GPCR-induced signaling (56). In conclusion, activated sensory nerves in the pancreas release SP, and the SP–NK-1R axis could stimulate many pathways like JAK/STAT, MAPK/ERK, and HER2/EGFR (mentioned before) signaling for oncogenesis and evolution of PDAC (56–59). While CGRP (encoded by the *calcitonin *gene) is a relatively novel neuropeptide first isolated in 1982, human-type CGRP is isolated from medullary thyroid carcinoma (60, 61). The release of CGRP by sensory neurons regulated by NGF *via* a cAMP/ras manner and activation of mitogen-activated protein kinase (MAPK) binds it to its target receptors CGRP1 and CGRP2, facilitating the concentration of cAMP followed by the activation of protein kinase A (PKA), which then unfolds K+ channels leading to the relaxation of smooth muscles in the vascular endothelium (62–64). Therefore, blood flow in PDAC is accelerated by the generation of CGRP, which is important for transporting trophic substances and modulation of metabolic plasticity. It has been published that CGRP has chemotherapy resistance in acute myeloid leukemia (AML) cell lines *via* the CGRP/CALCRL axis and promotes prostate tumor growth in murine models, possibly *via* ERKs/STAT3 signaling (65, 66). By the end of the article, there are few experimental articles about the specific impacts of CGRP and its receptors in PDAC. Hence, we promote a possible mechanism of sensory released CGRP based on the existing theories: secreted CGRP binds to CGRP1/CGRP2, activates cAMP/PKA signaling, K+ channel, and expansionary blood flow potentially accompanied with another bypass, which renders PDAC abundant nutrition, metabolic materials, and neural/metabolic plasticity. Hopefully, this article could provoke more related experiments to unveil the secretory characteristics of CGRP in PDAC. Taken together, neuropathic pain caused by PDAC induces sensory nerve impulses and then evokes TRPV1 to release neuropeptides, including CGRP and SP, which directly or indirectly activates individual signaling pathways for the tumorigenesis and evolution of PDAC. Recent research claims that the neuropathic pain of PDAC results from the abnormally expressed sonic hedgehog (sHH) signaling and is mediated by SP and CGRP in DRGs in an NGF-dependent manner (67). Furthermore, PanIN organoid colonies are directly promoted by sensory neurons *via* SP/NK-1R signaling and phosphorylated STAT3 (33, 68). Similarly, the NK-1R+ neuroendocrine cells in PanIN shows trophic effects to potentiate global organoid growth and provide developmental signals for NK-1R‐ cells to accelerate the growth rate of organoid colonies. Inversely, sensory denervation in KPCPdx1 mice greatly reduced the growth and progression of PanIN. Meanwhile, the subpopulation of NK-1R+ cells possibly correlated to impaired STAT3 phosphorylation and neuroendocrine cell maintenance (33). Interestingly, capsaicin purified from the pepper plant has a dose-dependent effect on its target TRPV1; the appropriate dose of capsaicin binds to TRPV1 and activates sensory nerves with the release of SP and CGRP, whereas an accumulating dose of capsaicin could achieve capsaicin desensitization which reversibly or permanently silences whole neurons for the treatment proposed (69, 70). According to these mechanisms, the application of TRPV1 antagonist (AG1529 has a partially inhibitory effect) has been approved to prevent the transition from acute pancreatitis to chronic pancreatitis (CP), along with the development of CP and inflammation-related pain in a murine model (71, 72). Similar treatments targeting the sensory innervation signaling in PDAC should be established only after they have been well tested to guarantee safety and validity.

### Parasympathetic nerves

Parasympathetic innervation in the pancreas originates from the dorsal motor nucleus of the vagus nerve as parasympathetic preganglionic fibers. It substitutes neurons with parasympathetic postganglionic neurons in intrapancreatic ganglia, which innervate the pancreas (36, 73–75). The parasympathetic nervous system is sorted into cholinergic nerves because its preganglionic and postganglionic neurons release Ach as neurotransmitters. When the vagal nerve is activated, the parasympathetic preganglionic nerve secrets Ach to activate parasympathetic postganglionic nerves *via* nAchRs about the ligand-gated ion channel family, and then postganglionic neurons also release Ach which binds to muscarinic acetylcholine receptors (mAchRs) at target tissues (76). There are five subtypes of mAchRs on target tissue cells, differing from M1 to M5 (encoded by *CHRM1* to *CHRM5*), and all five belong to the GPCR family (77, 78). It is illustrated that Ach, along with its acetylcholine receptor ligands, overexpresses several human cancers like colon, gastric, lung, and PDAC (44, 79–82). Cholinergic signaling induces an immune-suppressive microenvironment for the neoplastic part in PDAC to support tumor growth. Mechanistically, the Ach/nAchRs pathway impairs CD8+ T cell accumulation, attenuates IFN-γ generation in CD8+ T cells, and switches Th1/Th2 balance to Th2 guiding phenotype through both *in* *vivo *orthotopic PDAC model and *in* *vitro *high-performance liquid chromatography (83). The proximate discovery about cholinergic signaling reveals an aspect of the secret why PDAC is considered a “cold tumor” and lacks reactions to immunotherapy. In other words, diffuse cholinergic signaling renders PDAC a privilege to escape the immune system and indirectly mediate the microenvironment of PDAC to promote its growth. In addition, nicotine, the agonist of nAchRs, simultaneously suppresses GABA signaling and accumulates ARDB signaling to activate the SHH pathway collectively and then directly induces the self-renewal of pancreatic cancer stem cells, a mass of which initializes oncogenesis and the progression of PDAC (84). Moreover, clinical statistics display that overexpressed M3 is associated with pejorative development and unfavorable prognosis of PDAC patients (*n* = 58), in whom the cells are usually located at invasive tumor budding cells, metastatic lymph nodes, and parasympathetic nerve fibers (79). This article suggests that M3 should be a novel marker to predict the prognosis of PDAC. However, it seldom excavates the molecular mechanisms of the Ach/M3 axis in PDAC, and a small specimen leads to its limitation for clinical usage. Intriguingly, increasing publications also show that PSNS has recently decelerated cancer evolvement (40, 82, 85). Experiments in breast cancers reveal the innervation of SNS and PSNS, illustrating opposite effects in the tumor microenvironment. The denervation of sympathetic nerves and the neurostimulation of parasympathetic nerves in breast tumor decelerate the expression of immune checkpoint molecules probably mediated by M1 cholinergic mechanisms including PD-1, PD-L1, and FOXP3, which significantly suppress the anti-cancer immune response and become a potential treatment for breast cancer (40, 86, 87). However, whether parasympathetic nervous neurostimulation (M1-associated) also decumulates the immune checkpoint molecules in PDAC (like breast cancer) is not discovered up to the present, and the relational theoretical blank remains as both mystery and opportunity for researchers. As mentioned before, PSNS is also indicated to impair immune response by regulating immune cells to induce an immune-suppressive microenvironment for PDAC growth (83). Meanwhile, Renz and colleagues investigated the parasympathetic system’s specific effects and the possible mechanisms in PDAC. They performed subdiaphragmatic vagotomy on genetically engineered KC mice to disclose parasympathetic denervation in PDAC, which appeared to accelerate PDAC tumorigenesis and augment the expression of M1 under *KRAS *mutation. Along with vagotomy, utilizing the antagonist (pilocarpine) and the agonist (scopolamine) of M1 receptors has a similar and opposite result for cell viability and expression of M1. Mechanistically, cholinergic signaling suppresses PDAC oncogenesis and development in primary PDAC and metastatic lesions through the activation of M1-related signaling, then reduces the CD44+ CD133+-marked CSC subpopulation, and suppresses the downstream EGFR/MAPK and PI3K/AKT pathway, which indirectly and directly inhibits PDAC development, respectively (85). Overall, this article reveals that PSNS, the cholinergic nerves, suppress the initial PDAC. Still it did not clarify the process of cholinergic signaling activation and what form of M1-related signaling stimulates it downstream for restraining the development of PDAC. Given the existing evidence of neurotransmitters mediating the tumorous functions of automatic innervation in PDAC, we consider that activated cholinergic signaling secretes Ach through parasympathetic postganglionic neurons binding to M1 receptors on the membrane of target tissue cells, followed by the inhibition of EGFR/MAPK and AKT/PI3K signaling and the reduction of CSC subpopulation stimulated by Ach/M1 receptor axis. Furthermore, treatment with pilocarpine reveals a lower M1 expression, but scopolamine shows a higher M1 expression consistent with vagotomy, which suggests that the expression of M1 muscarinic receptors has a potential feedback loop with M1-related signaling activation.

To date, there is an extensive debate on the complicated role of the parasympathetic nervous system during the tumorigenesis of PDAC. Abundant innervations in the pancreas and complex cholinergic neurotransmitters and receptors result in the externally inconsistent conclusion of PSNS in PDAC. Here we propose a possible unified explanation for the two distinct opposite mechanisms of cholinergic signaling: tumorous plasticity of PDAC. PDAC, the most aggressive cancer, has incomparable adaption and high plasticity for its aggressive proliferation, growth, invasion, and metastasis. Take metabolic plasticity, for instance, PDAC could transfer its respiration manner from oxidative phosphorylation (OXPHOS)-dependent manner into aerobic glycolysis, leading to a style under a hypoxic microenvironment (emerging of the Warburg effect) (88); glycolysis and OXPHOS can also co-exist in the same lesions (89). Moreover, glycolytic PDAC cells even transform pyruvate in the mitochondria for OXPHOS when glycolysis is inhibited to promote energy and metabolites for proliferation and invasion (90). Taken together, we deem that the only famous principle about PDAC is that such aggressive cancer cells would take whatever it takes to invade and proliferate. Besides metabolic plasticity, angiogenesis, neurogenesis, immune suppression, systematic cell death (like apoptosis, autophagy, pyroptosis, and ferroptosis), chemo/radiotherapy resistance, *etc.*, generally confirm the proposed principle. The flexible plasticity weaves a complex and gigantic signaling network for tumor initialization and development. It is pretty arbitrary to consider that there is only one independent signaling pathway like PSNS for PDAC progression, which has a dichotomous “all or none” impact without any other possibility. We forecast that PSNS might have a plastic impact on PDAC, while the entire signaling network remains obscure and needs to be thoroughly excavated in PDAC. Moreover, targeting or suppressing associated signaling should be affirmatively verified and experimented with for clinical application in PDAC patients because the deformability and plasticity might switch the PDAC cells into more invasive types.

## Neuronal–metabolic interactions in PDAC

The latest research suggests the interaction between neuronal innervation and metabolic remodeling in PDAC (28, 91). Notably, metabolic plasticity renders PDAC cells to adapt to nutrient-deprived microenvironment *via* increasing scavenging pathways like autophagy, apoptosis, and reverse Warburg effects associated with pancreatic stellate cells (PSCs) or cancer-associated fibroblasts (CAFs) (92–94). Highly innervated PDAC has a strong interaction between PDAC cells and innervation from the microenvironment, and neurons also release amino acids (AA) as neurotransmitters, like D-serine, for long-term potentiation at the synapses or neurodevelopment like synapse maturation and axonal stabilization (95, 96). Given that serine is a non-essential AA and the little evidence about the metabolic association with tumor growth, the authors investigated the possibility of whether neurons have metabolic support to PDAC cells under a nutrient-deprived condition. In summary, this paper innovatively proves that peripheral axons secrete serine to metabolically sustain PDAC growth under a nutrient-deprived microenvironment (28).

### Metabolic reprogramming

**Figure 3:**
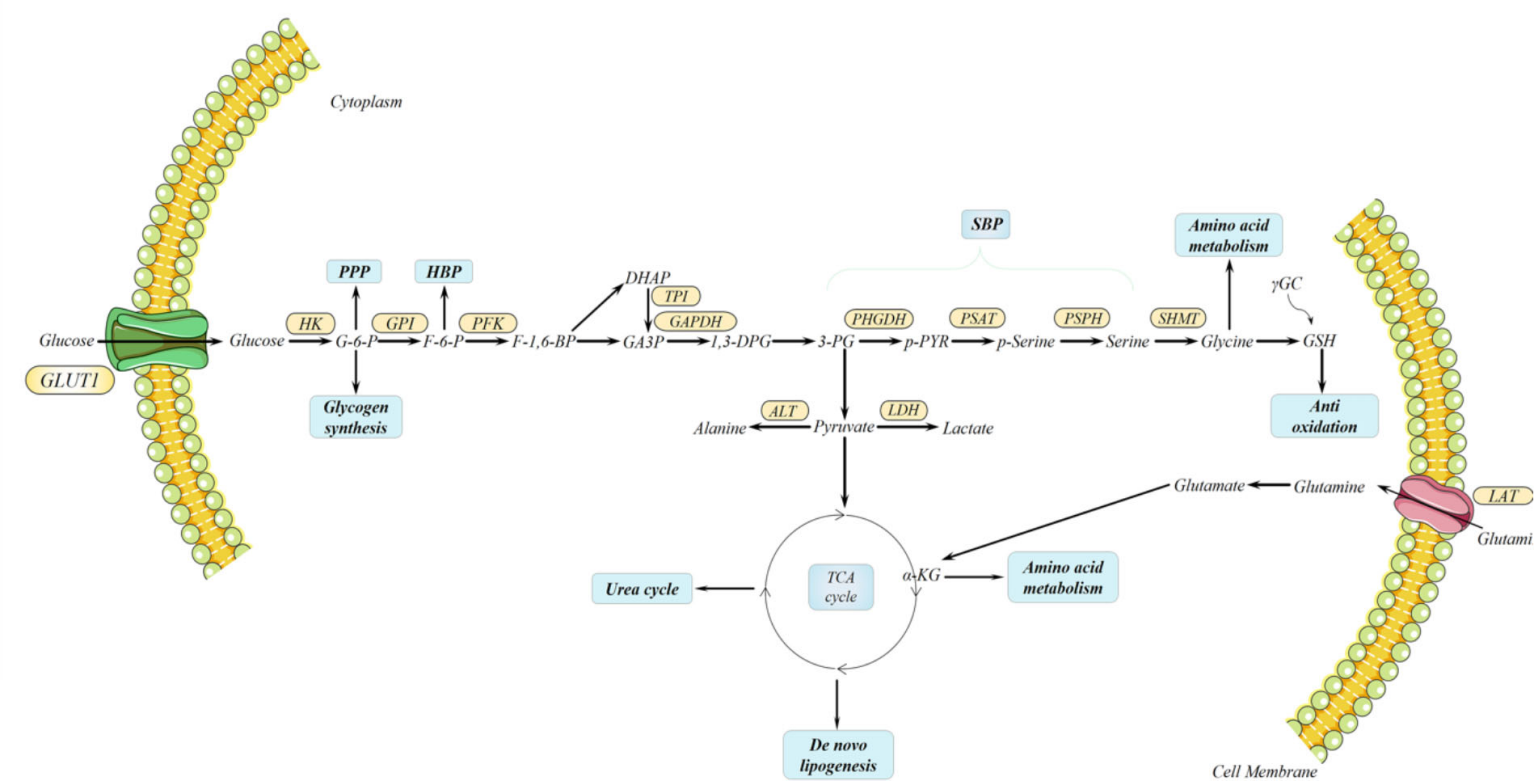
Metabolic rearrangements in pancreatic ductal adenocarcinoma (PDAC). In both primary and metastatic PDAC, metabolic rearrangements render PDAC extraordinary plasticity for the metabolites deprived. Among these rearrangements, mitochondrial respiration unifies several important bioenergetics; it produces precursors for lipid, amino acid, and nucleotide biosynthesizing and contributing to glutamine catabolism. Moreover, serine biosynthesis pathway is derived from glycolysis to produce serine for lipid synthesis, protein synthesis, and one-carbon cycle supporting nucleotide synthesis, methylation reactions, and antioxidant defense. Serine is catalyzed into glycine by serine hydroxymethyltransferase, which is converted to glutathione subsequently.

Aggressive cancer cells experience extensive metabolic rewiring during initial development.** **Dysregulated metabolism of glucose, lipid, and amino acid is confirmed in both primary and metastatic PDAC; those products and intermediates from those processes are essential for energy generation and material syntheses like cellular membranes, nucleotide, extracellular matrix (ECM), and cell cytoskeletons, which are fundamental for proliferation, invasion, and metastasis (12, 97–100). In PDAC, cancer cells uptake augmented glucose for metabolism and energy generation, which is utilized in positron emission tomography-computed tomography (101, 102). Glucose transfers into the cytoplasm through glucose transporter 1 (GLUT1) in the cell membrane, also highly expressed in PDAC associated with worse prognosis, and hexokinase catalyzes glucose into glucose-6-phosphate (G-6-P) where it divides into a bypass pentose phosphate pathway (PPP) contributing to the synthesis of DNA or RNA (103, 104). Another side path destination of G-6-P is synthesizing glycogen. During the course, glycogen synthase kinase 3 (GSK-3), an important kinase for glycogen synthesis, is reported to induce chemo-resistance for PDAC cells by sustaining the TopBP1/ATR/Chk1 DNA damage response pathway (105). Then, G-6-P is converted into fructose-6-phosphate (F-6-P) through the catalysis of phosphohexose isomerase, under the name of autocrine motility factor, phosphoglucose isomerase, and glucose-6-phosphate isomerase, which leads to another route, the hexosamine biosynthetic pathway (HBP). The end product of HBP is uridine diphosphate N-acetyl glucosamine (UDP-GlcNAc), generated by glucose, glutamine, and glucosamine and utilized in *O*-GlcNAcylation which is a post-translational modification, mainly glycosylation of serine and threonine (106). Moreover, HBP promotes the progression and survival pathways in PDAC and induces hyaluronan synthesis in the ECM. Furthermore, a study points out that they target glutamine-utilizing enzyme glutamine-fructose aminotransferase 1 *via* a small molecular glutamine analog, 6-diazo-5-oxo-I-norleucine, demonstrating reduced metastasis and self-renewal potential, along with decreased hyaluronan and collagen in the ECM, and sensitivity to immune checkpoint therapy (anti-PD1 therapy), thus resulting in better prognosis (107). HBP is significantly interlinked together with other metabolic pathways, including glucose, lipid, protein, and nucleotide, which exceedingly support the survival and progression of PDAC. Next, F-6-P is turned into 3-phosphoglycerate (3-PG) through the catalysis of a series of glycolytic enzymes, including phosphofructokinase (PFK), triose phosphate isomerase (TPI), *etc.* 3-PG directs to a shunt pathway, the serine biosynthesis pathway (SBP), employing 3-PG as a substrate for serine production. Such suggests that glycolytic intermediates could link to SBP from 3-PG and *vice versa*; serine can be catabolized into pyruvate. To start, 3-PG is transformed into 3-phosphohydroxypyruvate by phosphoglycerate dehydrogenase (PHGDH), which is the first enzyme in SBP, followed by phosphoserine aminotransferase 1 (PSAT1), thus inducing the transition to phosphoserine (p-serine). Subsequently, phosphoserine phosphatase (PSPH) mediates the generation of serine from p-serine, which is the last step of SBP. Serine, the second most abundant amino acid in human proteins, participates in many biosynthetic pathways, such as lipid synthesis, protein synthesis, and the one-carbon cycle, which supports nucleotide synthesis, methylation reactions, and antioxidant defense (108, 109). Interestingly, PHGDH, PSAT1, and PSPH overexpression is found in PDAC and correlated to shorter OS (110–112). When serine is limiting, augmented SBP, especially PHGDH, provides a growth advantage for tumor proliferation *via* sufficient serine in breast cancer and melanoma compared with normal environmental serine levels (113). Besides the metabolic aspect, SBP enzymes promote PDAC development by regulating molecular signaling. Specifically, PHGDH interlinks with translation initiation factors eIF4A1 and eIF4E while assembling translation initiation complex eIF4F to directly regulate protein synthesis, thus further promoting the progression and development of PDAC (110). In addition, overexpressed enzymes like PHGDH and PSAT1 promote the MYC/miR-494/enhancer of zeste homolog 2 (EZH2) feed-forward loop and are at least partially driven by MYC/ATF4 signaling in Burkitt lymphoma cells to sustain metabolic reprogramming. Notably, one carbon unit (including folate and methionine) produced from serine induces the production of S-adenosylmethionine, which facilitates the reaction of histone H3 trimethylation (H3K27me3), subsequently repressing miR-494 expression and thus decreasing the expression of MYC. MYC indirectly induces the methylation of H3K27me3 through regulating EZH2; the regulators combine into a feed-forward loop to sustain metabolic rearrangements (114). Clinically, the expression of PHGDH has been linked with lymph node metastasis in PDAC patients (115). Similarly, the selective loss of PSAT1 also abrogates migration, invasion, and metastasis in triple-negative breast cancer, suggesting that PSAT1 induces the migratory potential for metastasis without synthesizing serine (116). PSPH, the last enzyme in TSBP, also shows serine-expectant function *via* suppressing 2-hydroxyglutarate to liberate DNA 5-hydroxymethylcytosine and nuclear receptor NR4A1 expression to facilitate pro-oncogenic gene expression for melanoma growth and metastasis (117). Serine catabolism in the mitochondria is indicated by serine hydroxymethyltransferase 2 (SHMT2) to generate glycine and formate. Inversely, SHMT1 can also promote the cytosol transition from glycine to serine (118). Chemical pyrrolo[3,2-d]pyrimidine compounds targeting SHMT2 inhibitors have been investigated for serine catabolism. Small molecular compounds AGF291, AGF320, and AGF347 were established for antitumor efficiency toward lung, colon, and PDAC *in vitro*, respectively. AGF327 also demonstrates a potent effect *in vivo *models exerting a therapeutic potential for SHMT2 suppression for PDAC (119). Afterward, glycine is used to synthesize glutathione (GSH); high levels of GSH have an anti-apoptosis function for chemotherapy resistance in PDAC, and GSH/GSSG ratios regulate reactive oxygen species damage (120). Following on the heels of 3-PG, pyruvate is produced from a three-step catalytic reaction based on 3-PG, which is the initial alanine synthesis. The reversible reaction of converting pyruvate to alanine catalyzes alanine aminotransferase 1, also known as glutamate pyruvic transaminase, in the cytoplasm and *vice versa*. Finally, pyruvate enters the mitochondria for the tricarboxylic acid (TCA) cycle or is directly catalyzed by lactate dehydrogenase for glycolysis in the cytoplasm, severally. The TCA cycle in the mitochondria unifies the urea cycle, *de novo *lipogenesis, and amino acid metabolism *via* its intermediates. Among the intermediates, citrate is transformed into acetyl-coenzyme A *via* ATP-citrate lyase and finally produces fatty acid for proliferative cellular membranes and energy production signaling molecules’ second messenger generation. In addition, fumarate, as an intermediate from the TCA and urea cycles, acts as the bridge between the two cycles. Moreover, another nutrient fuel for biosynthesis other than glucose glutamine, a dispensable amino acid, enters cells through the alanine/serine/cysteine-preferring transporter 2 (ASCT2) and turns into glutamate *via* glutaminase, which is subsequently transferred into α-ketoglutarate (α-KG) *via* ALT2 as carbon donors (121). Notably, glutamine also provides nitrogen for other non-essential amino acid synthesis like serine, glycine, alanine, *etc.* (**Figure 3**).

Until recently, increasing evidence supports the dynamically metabolic transition in metastatic cancers during the changing microenvironment. Interestingly, an article claims that aggressive cancer adjusts its metabolism to adapt to a shifty microenvironment and support growth at every stage during its metastatic cascade (98). Moreover, the authors define two novel concepts about metabolic adjustment: metabolic plasticity and flexibility. Notably, metabolic plasticity refers to the same metabolite used by metastatic cells during its different stages of metastasis, and metabolic flexibility (established on nutrient flexibility) describes different metabolites that can all fulfill the exact metabolic requirement of metastatic cascade in metastatic cells (98). Notably, PDAC, the most aggressive cancer, undergoes complicated metabolic rearrangements. Although metastasis in PDAC is a rare process, distant metastasis and cachexia of PDAC are significant causes of the death of cancer patients. Understanding the metabolic reprogramming of PDAC cells in every stage during its metastasis may promote measurements to destroy its metabolic process *via* targeting the metabolic vulnerabilities for potential therapy (**Table 1**).

**Table 1:** Clinical evidence of metabolic expression for detection in pancreatic ductal adenocarcinoma (PDAC).

Molecules	Expression	Source	References	Possible pathways
***Glycolysis***				
Glucose	Up	Temporal fasting blood glucose (FBG) profiles before diagnosis (*n* = 219), for resected volume and grade (*n* = 526), and for long-term FBG data (*n* = 103)	(122)	Participating in glycolysis, oxidative phosphorylation (OXPHOS), and tumorigenesis (103, 123)
Pyruvate	Up	RNA sequencing from laser-captured microdissected human PDAC compared with normal pancreatic tissue (*n* = 21)	(124)	Participating in glycolysis, OXPHOS, and alanine (125)
Lactate	Up	Pancreatic juice from PDAC patients (*n* = 79) and non-PDAC patients (*n* = 27)	(126)	Form acidified environment for PDAC invasion and epigenomic reprogramming of cancer-associated fibroblasts (127, 128)
Glut	Up	Immunohistochemical staining for PDAC resected samples compared with adjacent normal pancreatic tissue (*n* = 39)	(104)	Transporting glucose into the cytoplasm, associated with tumor grade and high density of PD-1^+^ T cells, and predicting poor prognosis (104, 126)
HK	Up	Gene dataset of resected tumors from primary and metastatic sites of PDAC patients (*n* = 143)	(129)	Catalyzing glucose into G-6-P and regulating lactate production to promote the growth of PDAC (129, 130)
GPI	Up	Immunohistochemical staining of paraffin sections from paired PDAC and control patients (*n* = 13)	(131)	Promoting the catalysis of G-6-P into F-6-P and enhancing the metastasis of PDAC (131)
PFK	Up	GeneChip hybridization of paired normal and tumor specimens from PDAC patients (*n* = 36)	(132)	Catalyzing F-6-P into F-1,6-BP and involved in localized ATP supply at the plasma membrane for poor prognosis (126)
LDH	Up	Immunohistochemical staining of LDHB on resected PDAC tumors (*n* = 59)	(133)	Catalyzing pyruvate into lactate and demonstrating resistance to therapy with gemcitabine (134)
TPI	Up	Gene dataset of primary PDAC tumors compared with normal pancreas and in metastatic PDAC compared with primary tumors (*n* = 143)	(129)	Inducing catalysis between DHAP and GA3P but showing no correlation with PDAC progression (135)
***Pyruvate to alanine***				
Alanine	Down	Individual amino acid profiles of PDAC patients (*n* = 12) and healthy control subjects (*n* = 12)	(136)	Participating in protein synthesis and maintaining compartmentalized pyruvate homeostasis (137)
ALT	Up	Blood samples obtained from every PDAC patient in 10 years (*n* = 1787)	(138)	Inducing the catalysis of alanine from pyruvate and predicting the clinical outcome of PDAC patients (139)
Asparagine synthetase	Up	Immunohistochemistry of tumor samples in oral squamous cell carcinoma (OSCC) patients (*n* = 86); also confirmed by The Cancer Genome Atlas OSCC cohort (*n* = 279)	(140)	Remarkably associating with PNI^+^ in cancer cells (140)
***SBP***				
Serine	Undifferentiated	Individual amino acid profiles of PDAC patients (*n* = 12) and healthy control subjects (*n* = 12)	(136)	Participating in protein synthesis and generation of glycine (28)
PHGDH	Up	Immunohistochemical analysis of PDAC patients (*n* = 24) compared with normal adjacent tissues (*n* = 4)	(110)	Catalyzing 3-PG into *p-*PYR and interacting with eIF4A1 and eIF4E for the development of PDAC (110, 112, 141)
PSAT	Up	Genome expression datasets of PDAC patients (*n* = 119) compared with normal subjects (*n* = 199)	(111)	Catalyzing *p-*PYR into *p-*serine and involved in the diagnosis and prognosis of PDAC (111, 112)
PSPH	Up	Mass spectrometry for protein abundance from four cell lines, but blank in PDAC patients (BxPC3M1, BxPC3, Panc1, and MiaPaca2)	(112)	Catalyzing *p-*serine into serine and promote cancer progression (112, 142)
***Serine to glycine***				
SHMT	Up	Immunohistochemical analysis of PDAC patients (*n* = 24) compared with normal adjacent tissues (*n* = 4)	(110)	Catalyzing the reversible interconversion of serine to glycine and promoting the proliferation and colony formation of PDAC (110)
Glycine	Undifferentiated	Individual amino acid profiles of PDAC patients (*n* = 12) and healthy control subjects (*n* = 12)	(136)	Participating in protein synthesis and generation of GSH (28)

### Neuronal–metabolic interaction through serine

**Figure 4:**
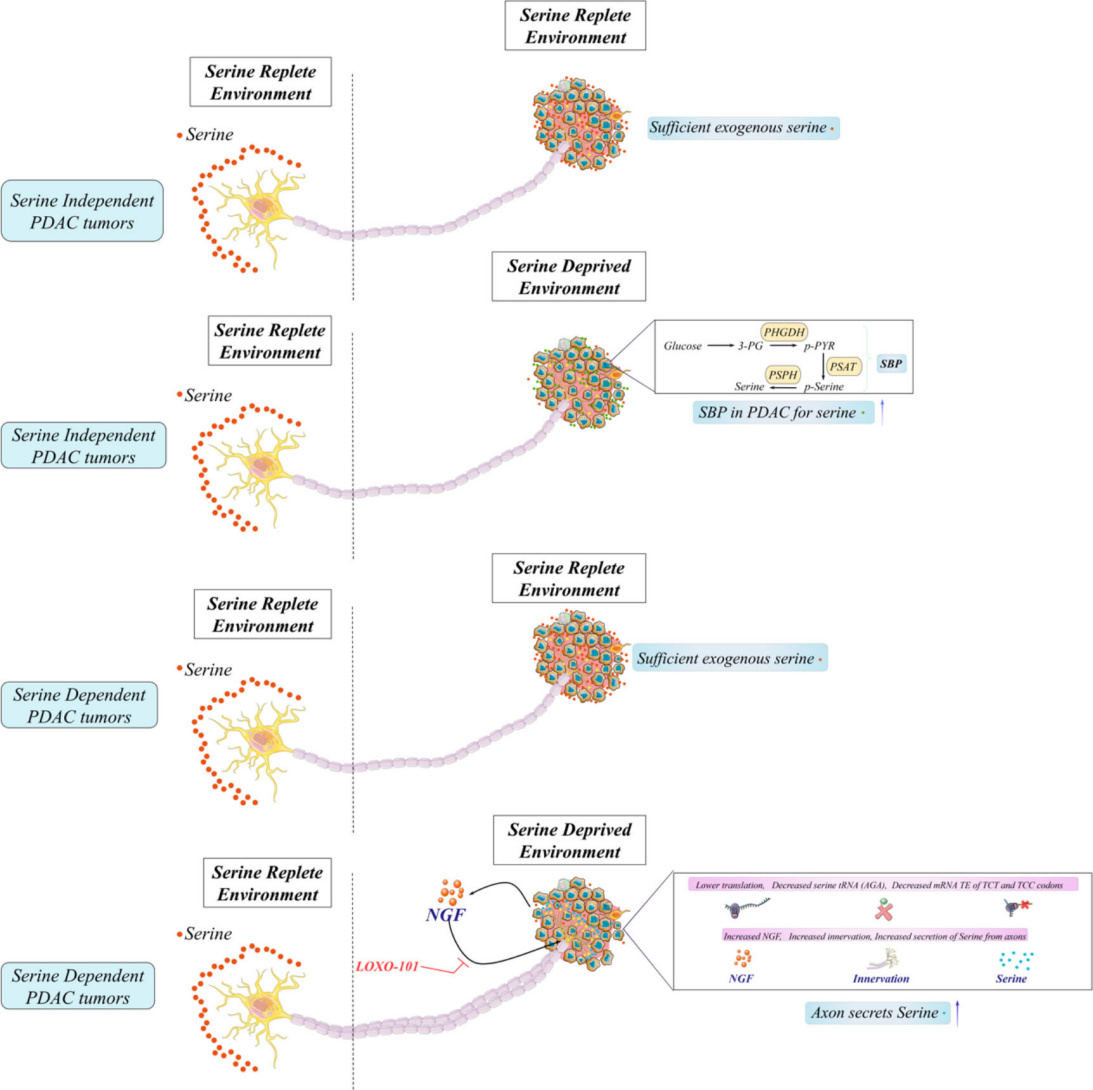
Neural and metabolic interactions in pancreatic ductal adenocarcinoma (PDAC). A large fraction of human PDAC cells lack SBP enzymes (PHGDH and PSAT-1) and cannot generate serine, and other pathways like autophagy and pancreatic stellate cells cannot generate sufficient levels of serine for growth in PDAC cells under serine deprivation. Therefore, these PDAC cells which must import serine for growth are exogenous serine-dependent PDAC. Moreover, those with SBP enzymes (PHGDH and PSAT-1) for the generation of serine from glucose are exogenous serine-independent PDAC. To date, neurons could directly promote metabolic support (releasing serine) for PDAC cells under the serine-deprived conditions in PDAC.

It has been accepted that PDAC has a strong tendency for innervation and abundant PNI. These features indicate the worse prognosis of PDAC (143). Early major research about neuron–tumor interlinks points tumor-inducing neurons in PDAC (144). Neurons in PDAC express abundant neurotrophic factors, including NGF, GDNF, artemin, and neuronal chemokines, promoting the malignancy of PDAC *via* signaling activation (145). As we described earlier, the New York research team identified the unique group of neoplastic neuroendocrine cells in PanINs of the mouse and human models. Neuropeptide receptor NK1-R is detected in the cells, and NK-1R^+^ cells functionally promote PanIN nutrition utilization, thus assisting PanIN organoid growth. Furthermore, denervation of sensory neurons in murine PDAC reduces NK-1R^+^ cell number and retards PanIN-PDAC progression (33). In this publication, they believe that sensory neurons facilitate PDAC formation and metabolic signaling *via* the SP–NK1R–STAT3 pathways. To date, other direct shreds of evidence of neurons and cancer development are discovered in PDAC from the metabolic aspect (28, 146). Although PSCs and macrophages in TME can supply PDAC with some metabolites or nutrition, it is insufficient for PDAC to get growth requirements with such metabolites (147). Thus, 5-HT (serotonin), a neuro-regulator, grows in PDAC tissues and cell lines compared with normal pancreas. Incubation of PDAC with serotonin or activator of HTR2B renders the proliferative capacity and inhibition of apoptosis. Moreover, serotonin activation leads to a complex of HTR2B-LYN-p85 that activates PI3K–Akt– mTOR signaling and then enhances the Warburg effect (glycolysis) *via* overexpressed MYC and HIF-1α. The neurotransmitter serotonin released by axons affects metabolic rearrangements, including glycolytic flux, PPP, and HBP, through the expression of enzymes (146). This work suggests that neurotransmitters enable signaling pathways in PDAC and then incite metabolic rearrangements, influencing the malignancy or survival of PDAC afterward. To sum up, the previous research connects neural–metabolic crosstalk in PDAC through chemokines, neurotrophins, and neurotransmitters to investigate the role of innervation during PDAC development. Innovatively, Hindson and colleagues hypothesized that neurons could directly promote metabolic support for PDAC cells under nutrient deprivation in PDAC (91). To assess, micro-fluidic devices that could isolate axons from the neuronal body of DRGs were applied to stimulate an *in vivo* situation where PDAC cells in nutrient-deprived TME are only exposed to peripheral axons, but not the neuronal bodies (148). Surprisingly, the detection of the conditional axonal media exhibits AA levels, including serine and glycine, suggesting that axons release AA into a nutrient-poor environment. There is a heterogeneity of human PDAC about their dependence on exogenous serine. Approximately 40% of human PDAC cell lines concretely depend on exogenous serine for proliferation due to a lack of expression or ability to augment PHGDH and PSAT1, while mouse PDAC cells can promote serine synthesis and are insensitive to serine starvation. However, the repressed growth of PDAC could be rescued by a co-culture with axons in the microfluidic devices (149). Mechanistically, they noticed reduced mitochondrial activity in serine-dependent PDAC under serine deprivation and a rapidly increased oxygen consumption rate (OCR). This change is similar to utilizing an inhibitor of protein synthesis, cycloheximide (150), which indicates that serine starvation selectively suppresses the mRNA translation rates for protein synthesis, causing decreased mitochondrial activity and increased OCR. More specifically, the authors explored the concrete effects through destabilized green fluorescent proteins (151), which replace all serine codons with one of the six serine codons. Among them, the translation efficiency of TCT and TCC is repressed because of ribosome stalling as revealed by ribosome profiling (152). Interestingly, NGF is intensely expressed by serine-dependent PDAC cells under serine deprivation because the selective translation allows the synthesis of TCC and TCT-low coded proteins, exhibiting enhanced nerve infiltration for serine and glycine secretion to restore PDAC growth. These observations are consistent with *in vivo* models. Exogenous serine-dependent PDAC under serine-starved conditions shows a significantly smaller tumor mass, accelerating NGF secretion and accumulating sympathetic and sensory innervation. In treating mice with LOXO-101, the NGF receptor inhibitor adversely reduces tumor mass and innervation under a serine-free diet compared with the serine-free-diet mice or control diet with LOXO-101 mice (153). Moreover, PDAC tumors with augmented levels of PHGDH typically have attenuated NGF, tumor innervation, and shorter OS, which is also consistent with other results (28). In summary, this article successfully testifies the metabolic assistance of neurons in PDAC, rendering great metabolic plasticity *via* neural innervation, which also hints us to concentrate on other aspects in TME for metabolic arrangements. However, as the first published document implicating the function of serine and innervation, there are still unclear aspects of this article, mainly about the mechanisms allowing neurons to release amino acid to support PDAC cells metabolically, mechanisms about the selective translation efficiency of codons, and why PDAC would lose the ability to synthesize serine? More importantly, clinical transformation based on the observed phenomenon would promote novel therapeutic potential targeting PDAC metabolic and neural crosstalk for PDAC patients (**Figure 4**).

Recently, some documents gave an overview on serine and other metabolites in neural–cancer progress (154). Serine is considered to not only facilitate cancer progress but also maintain neurites’ formation—for example, NGF induces the phosphorylation of RhoA on serine 188 and reduces RhoA–Rho-related kinase for neurite growth (155); another *in vitro* experiment declaims that NGF-associated phosphorylated STAT3 at serine (727) is involved in neurite growth (156). Apart from serine, additional metabolites are associated with neural innervation in cancers. For a start, solute carrier family 2 member 3 (SLC2A3), asparagine, and asparagine synthetase are all remarkably expressed in PNI+ cancer. Overexpressed SLC2A3, increased glucose, and reduced vitamin C indicate a diminished OS in colorectal cancer patients and AML patients (157, 158). However, the concrete part of SLC2A3 in neural–metabolic crosstalk is not excavated. The immunohistochemistry of OSCC clinical study (*n* = 86), together with The Cancer Genome Atlas database (*n* = 267), presents that asparagine synthetase is notably added in PNI+ tumors, and L-asparagine is the only amino acid able to indicate PNI with satisfactory sensitivity and specificity (140). Regrettably, these studies fail to trench the concrete mechanisms of the metabolic–neural functions during cancer development. Banh and colleagues introduce an excellent method to excavate metabolic–neural interactions in cancer through microfluidic devices to isolate neurons and cancer cells in nutrition-deprived conditions to stimulate an *in vivo* environment, and we believe that utilizing the unique method could elaborate the specific mechanisms of metabolic–neural interlinks during cancer evolution, especially metabolites like serine and glycine.

## Conclusion

To sum up, we first summarized the epidemiology of PDAC, especially risk factors and clinical symptoms, and then introduced a classification for PDAC and related genomic alterations. Notably, the pancreas is a highly innervated organ, so we also discussed three different nerve innervation and pro/anti-oncogenic functions in PDAC. Lastly, we mainly reviewed metabolic rearrangements in PDAC and commented on a recent article about neural and metabolic crosstalk. This review concludes the relationship between innervation and PDAC and also recommends a novel method to excavate metabolic–neural interactions in cancer through microfluidic devices to isolate neurons and cancer cells introduced by Banh and colleagues. We believe that a more direct proof of metabolic–neural crosstalk in PDAC remains unclear, especially metabolites like amino acids. Hopefully, this article could further explore the pathogenic mechanisms of PDAC and then induce the transformation of detection and treatment for PDAC considering the innervation.

## Author contributions

RC and MD contributed to the conception and design of the study. LC wrote the first draft of the manuscript. YX and MD wrote sections of the manuscript and provided the critical revisions. All authors contributed to the article and approved the submitted version.

## Funding

This article is funded by following projects: Special Medical and Health Personnel Program of Jilin Province Finance Department (2019SCZT015) and Health Technology Innovative Program of Jilin Province (2018J053).

## Conflict of interest

The authors declare that the research was conducted in the absence of any commercial or financial relationships that could be construed as a potential conflict of interest.

## Publisher’s note

All claims expressed in this article are solely those of the authors and do not necessarily represent those of their affiliated organizations, or those of the publisher, the editors and the reviewers. Any product that may be evaluated in this article, or claim that may be made by its manufacturer, is not guaranteed or endorsed by the publisher.

## Glossary

**Table:**

2-HG	2-hydroxyglutarate
3-PG	3-phosphoglycerate
3-pPYR	3-phosphohydroxypyruvate
5hmC	5-hydroxymethylcytosine
AA	amino acid
ACC	acinar cell carcinoma
Ach	acetylcholine
ACLY	ATP-citrate lyase
ADM	acinar to ductal metaplasia
ADRB	β-adrenergic receptors
ALT1	Alanine aminotransferase 1
AML	acute myeloid leukemia
ANS	autonomic nervous system
AP	acute pancreatitis
ASCT2	alanine/serine/cysteine-preferring transporter 2
BDNF	brain derived neurotrophic factor
BL	Burkitt lymphoma
CAFs	cancer-associated fibroblasts
CGRP	calcitonin gene-related peptide
CHX	cycloheximide
CNS	central nerve system
CoA	acetyl-coenzyme A
CP	chronic pancreatitis
DON	6-diazo-5-oxo-I-norleucine
DRG	dorsal root ganglia
ECM	extracellular matrix
EZH2	enhancer of zeste homolog 2
F-6-P	fructose-6-phosphate
G-6-P	glucose-6-phosphate
GDNF	glial cell line-derived neurotrophic factor
GFAT1	glutamine-fructose aminotransferase 1
GFPs	green fluorescent proteins
GLUT1	glucose transporter 1
GPCR	G-protein coupled receptors
GPT	glutamate pyruvic transaminase;
GSH	glutathione
GSK-3	glycogen synthase kinase 3
H3K27me3	H3 trimethylation;
HBP	hexosamine biosynthetic pathway
HK	hexokinase
HPLC	high-performance liquid chromatography
IPMN	intraductal papillary mucinous neoplasm
ISO	isoproterenol
ITPN	intraductal tubulopapillary neoplasm
KC	LSL-*Kras* ^+/LSL-G12D^;*Pdx1*-Cre
KPC	LSL-*Kras* ^+/LSL-G12D^;LSL-*Trp53* ^+/R172H^;*Pdx1*-Cre
LDH	lactate dehydrogenase
LGICs	ligand-gated ion channels
mAchRs	muscarinic acetylcholine receptors
MAPK	mitogen-activated protein kinase
MCN	mucinous cystic neoplasm
*MMP2*	*matrix metalloprotease 2*
nAchRs	nicotinic acetylcholine receptors
NB	β-blocker
NBB	no β blockers
NE	norepinephrine
NG	nodose ganglia
NGF	neural growth factors
NK-1R	*n*eurokinin receptor 1
NSBB	non-selective β-blockers
NT	neurotrophins
NTF3	neurotrophic factor 3
OCR	oxygen consumption rate
OS	overall survival
OXPHOS	oxidative phosphorylation
PanNEN	pancreatoblastoma, pancreatic neuroendocrine neoplasm
PCSCs	pancreatic cancer stem cells
PDAC	pancreatic ductal adenocarcinoma
PET-CT	positron emission tomography computed tomography
PFK	phosphofructokinase
PHGDH	phosphoglycerate dehydrogenase
PHI	phosphohexose isomerase
PKA	protein kinase A
PNI	perineural invasion
PPP	pentose phosphate pathway
*Ppt-a*	*pre-pro-tachykinin-A*
PSAT1	phosphoserine aminotransferase 1
PSCs	pancreatic satellite cells
p-serine	phosphoserine
PSNS	parasympathetic nervous system;
PSPH	phosphoserine phosphatase
PTM	post-translational modification
ROS	reactive oxygen species
SAM	S-adenosylmethionine
SB1B	β-1 selective blocker
SBP	serine biosynthesis pathway
sHH	sonic hedgehog
SHMT2	serine hydroxymethyltransferase 2
SP	substance P
SPN	solid pseudopapillary neoplasm
TCA	tricarboxylic acid
TME	tumor microenvironment
TNBC	triple negative breast cancer
TPI	triose phosphate isomerase
TRPV1	transient receptor potential vanilloid 1
UDP-GlcNAc	uridine diphosphate N-acetyl glucosamine
α-KG	α-ketoglutarate

## References

[1] Siegel RL Miller KD Jemal A. Cancer statistics, 2020. CA: Cancer J Clin (2020) 70(1):7–30. doi: 10.3322/caac.21590 31912902

[2] Flavahan WA Gaskell E Bernstein BE. Epigenetic plasticity and the hallmarks of cancer. Science (2017) 357(6348):eaal2380. doi: 10.1126/science.aal2380 28729483 PMC5940341

[3] Vitale I Shema E Loi S Galluzzi L. Intratumoral heterogeneity in cancer progression and response to immunotherapy. Nat Med (2021) 27(2):212–24. doi: 10.1038/s41591-021-01233-9 33574607

[4] Rahib L Smith BD Aizenberg R Rosenzweig AB Fleshman JM Matrisian LM. Projecting cancer incidence and deaths to 2030: the unexpected burden of thyroid, liver, and pancreas cancers in the united states. Cancer Res (2014) 74(11):2913–21. doi: 10.1158/0008-5472.CAN-14-0155 24840647

[5] Siegel RL Miller KD Fuchs HE Jemal A. Cancer statistics, 2021. CA: Cancer J Clin (2021) 71(1):7–33. doi: 10.3322/caac.21654 33433946

[6] Zaccari P Cardinale V Severi C Pedica F Carpino G Gaudio E. Common features between neoplastic and preneoplastic lesions of the biliary tract and the pancreas. World J Gastroenterol (2019) 25(31):4343–59. doi: 10.3748/wjg.v25.i31.4343 PMC6710182 31496617

[7] Felsenstein M Noë M Masica DL Hosoda W Chianchiano P Fischer CG. IPMNs with co-occurring invasive cancers: neighbours but not always relatives. Gut (2018) 67(9):1652–62. doi: 10.1136/gutjnl-2017-315062 PMC10489026 29500184

[8] Scarpa A Real FX Luchini C. Genetic unrelatedness of co-occurring pancreatic adenocarcinomas and IPMNs challenges current views of clinical management. Gut (2018) 67(9):1561–3. doi: 10.1136/gutjnl-2018-316151 29661802

[9] Mostafa ME Erbarut-Seven I Pehlivanoglu B Adsay V. Pathologic classification of "pancreatic cancers": current concepts and challenges. Chin Clin Oncol (2017) 6(6):59. doi: 10.21037/cco.2017.12.01 29307199

[10] Mizrahi JD Surana R Valle JW Shroff RT. Pancreatic cancer. Lancet (London England) (2020) 395(10242):2008–20. doi: 10.1016/S0140-6736(20)30974-0 32593337

[11] Bachmann J Heiligensetzer M Krakowski-Roosen H Büchler MW Friess H Martignoni ME. Cachexia worsens prognosis in patients with resectable pancreatic cancer. J gastrointestinal Surg (2008) 12(7):1193–201. doi: 10.1007/s11605-008-0505-z 18347879

[12] Cao L Wu J Qu X Sheng J Cui M Liu S. Glycometabolic rearrangements–aerobic glycolysis in pancreatic cancer: causes, characteristics and clinical applications. J Exp Clin Cancer Res (2020) 39(1):267. doi: 10.1186/s13046-020-01765-x 33256814 PMC7708116

[13] Grossberg AJ Chu LC Deig CR Fishman EK Hwang WL Maitra A. Multidisciplinary standards of care and recent progress in pancreatic ductal adenocarcinoma. CA: Cancer J Clin (2020) 70(5):375–403. doi: 10.3322/caac.21626 32683683 PMC7722002

[14] Arslan AA Helzlsouer KJ Kooperberg C Shu XO Steplowski E Bueno-de-Mesquita HB. Anthropometric measures, body mass index, and pancreatic cancer: a pooled analysis from the pancreatic cancer cohort consortium (PanScan). Arch Internal Med (2010) 170(9):791–802. doi: 10.1001/archinternmed.2010.63 20458087 PMC2920035

[15] Bosetti C Lucenteforte E Silverman DT Petersen G Bracci PM Ji BT. Cigarette smoking and pancreatic cancer: an analysis from the international pancreatic cancer case-control consortium (Panc4). Ann Oncol (2012) 23(7):1880–8. doi: 10.1093/annonc/mdr541 PMC3387822 22104574

[16] Lowenfels AB Maisonneuve P Cavallini G Ammann RW Lankisch PG Andersen JR. Pancreatitis and the risk of pancreatic cancer. international pancreatitis study group. New Engl J Med (1993) 328(20):1433–7. doi: 10.1056/NEJM199305203282001 8479461

[17] Hu C Hart SN Polley EC Gnanaolivu R Shimelis H Lee KY. Association between inherited germline mutations in cancer predisposition genes and risk of pancreatic cancer. Jama (2018) 319(23):2401–9. doi: 10.1001/jama.2018.6228 PMC6092184 29922827

[18] Petersen GM. Familial pancreatic cancer. Semin Oncol (2016) 43(5):548–53. doi: 10.1053/j.seminoncol.2016.09.002 PMC5234085 27899186

[19] Lowenfels AB Maisonneuve P DiMagno EP Elitsur Y Gates LK Jr. Perrault J. Hereditary pancreatitis and the risk of pancreatic cancer. international hereditary pancreatitis study group. J Natl Cancer Institute (1997) 89(6):442–6. doi: 10.1093/jnci/89.6.442 9091646

[20] Giardiello FM Brensinger JD Tersmette AC Goodman SN Petersen GM Booker SV. Very high risk of cancer in familial peutz-jeghers syndrome. Gastroenterology (2000) 119(6):1447–53. doi: 10.1053/gast.2000.20228 11113065

[21] Brune KA Lau B Palmisano E Canto M Goggins MG Hruban RH. Importance of age of onset in pancreatic cancer kindreds. J Natl Cancer Institute (2010) 102(2):119–26. doi: 10.1093/jnci/djp466 PMC2808346 20068195

[22] Schmidt-Hansen M Berendse S Hamilton W. Symptoms of pancreatic cancer in primary care: A systematic review. Pancreas (2016) 45(6):814–8. doi: 10.1097/MPA.0000000000000527 26495795

[23] Bapat AA Hostetter G Von Hoff DD Han H. Perineural invasion and associated pain in pancreatic cancer, nature reviews. Cancer (2011) 11(10):695–707. doi: 10.1038/nrc3131 21941281

[24] Liebl F Demir IE Mayer K Schuster T Dʼ;Haese JG Becker K. The impact of neural invasion severity in gastrointestinal malignancies: a clinicopathological study. Ann Surg (2014) 260(5):900–7; discussion 907-8. doi: 10.1097/SLA.0000000000000968 25379860

[25] Biankin AV Waddell N Kassahn KS Gingras MC Muthuswamy LB Johns AL. Pancreatic cancer genomes reveal aberrations in axon guidance pathway genes. Nature (2012) 491(7424):399–405. doi: 10.1038/nature11547 23103869 PMC3530898

[26] Harris NLE Vennin C Conway JRW Vine KL Pinese M Cowley MJ. SerpinB2 regulates stromal remodelling and local invasion in pancreatic cancer. Oncogene (2017) 36(30):4288–98. doi: 10.1038/onc.2017.63 PMC5537606 28346421

[27] Pavlides S Tsirigos A Vera I Flomenberg N Frank PG Casimiro MC. Transcriptional evidence for the "Reverse warburg effect" in human breast cancer tumor stroma and metastasis: similarities with oxidative stress, inflammation, alzheimer's disease, and "Neuron-glia metabolic coupling". Aging (2010) 2(4):185–99. doi: 10.18632/aging.100134 PMC2881509 20442453

[28] Banh RS Biancur DE Yamamoto K Sohn ASW Walters B Kuljanin M. Neurons release serine to support mRNA translation in pancreatic cancer. Cell (2020) 183(5):1202–1218.e25. doi: 10.1016/j.cell.2020.10.016 33142117 PMC8100789

[29] Borden P Houtz J Leach SD Kuruvilla R. Sympathetic innervation during development is necessary for pancreatic islet architecture and functional maturation. Cell Rep (2013) 4(2):287–301. doi: 10.1016/j.celrep.2013.06.019 23850289 PMC3740126

[30] Bockman DE. Nerves in the pancreas: what are they for? Am J Surg (2007) 194(4, Supplement):S61–4. doi: 10.1016/j.amjsurg.2007.05.028

[31] Waxenbaum JA Reddy V Varacallo M. Anatomy, autonomic nervous system. In: StatPearls. Treasure Island (FL: StatPearls Publishing LLC. (2021). 30969667

[32] Yi SQ Miwa K Ohta T Kayahara M Kitagawa H Tanaka A. Innervation of the pancreas from the perspective of perineural invasion of pancreatic cancer. Pancreas (2003) 27(3):225–9. doi: 10.1097/00006676-200310000-00005 14508126

[33] Sinha S Fu YY Grimont A Ketcham M Lafaro K Saglimbeni JA. PanIN neuroendocrine cells promote tumorigenesis *via* neuronal cross-talk. Cancer Res (2017) 77(8):1868–79. doi: 10.1158/0008-5472.CAN-16-0899 PMC5471615 28386018

[34] Magnon C Hall SJ Lin J Xue X Gerber L Freedland SJ. Autonomic nerve development contributes to prostate cancer progression. Science (2013) 341(6142):1236361. doi: 10.1126/science.1236361 23846904

[35] Stopczynski RE Normolle DP Hartman DJ Ying H DeBerry JJ Bielefeldt K. Neuroplastic changes occur early in the development of pancreatic ductal adenocarcinoma. Cancer Res (2014) 74(6):1718–27. doi: 10.1158/0008-5472.CAN-13-2050 PMC4036226 24448244

[36] Gasparini G Pellegatta M Crippa S Lena MS Belfiori G Doglioni C. Nerves and pancreatic cancer: New insights into a dangerous relationship. Cancers (2019) 11(7). doi: 10.3390/cancers11070893 PMC6678884 31248001

[37] Tan X Sivakumar S Bednarsch J Wiltberger G Kather JN Niehues J. Nerve fibers in the tumor microenvironment in neurotropic cancer-pancreatic cancer and cholangiocarcinoma. Oncogene (2021) 40(5):899–908. doi: 10.1038/s41388-020-01578-4 33288884 PMC7862068

[38] Kim-Fuchs C Le CP Pimentel MA Shackleford D Ferrari D Angst E. Chronic stress accelerates pancreatic cancer growth and invasion: a critical role for beta-adrenergic signaling in the pancreatic microenvironment. Brain behavior Immun (2014) 40:40–7. doi: 10.1016/j.bbi.2014.02.019 PMC4102665 24650449

[39] Guo K Ma Q Li J Wang Z Shan T Li W. Interaction of the sympathetic nerve with pancreatic cancer cells promotes perineural invasion through the activation of STAT3 signaling. Mol Cancer Ther (2013) 12(3):264–73. doi: 10.1158/1535-7163.MCT-12-0809 23288783

[40] Kamiya A Hayama Y Kato S Shimomura A Shimomura T Irie K. Genetic manipulation of autonomic nerve fiber innervation and activity and its effect on breast cancer progression. Nat Neurosci (2019) 22(8):1289–305. doi: 10.1038/s41593-019-0430-3 31285612

[41] Renz BW Takahashi R Tanaka T Macchini M Hayakawa Y Dantes Z. β2 adrenergic-neurotrophin feedforward loop promotes pancreatic cancer. Cancer Cell (2018) 33(1):75–90.e7. doi: 10.1016/j.ccell.2017.11.007 29249692 PMC5760435

[42] Kopp JL von Figura G Mayes E Liu FF Dubois CL Morris JPT. Identification of Sox9-dependent acinar-to-ductal reprogramming as the principal mechanism for initiation of pancreatic ductal adenocarcinoma. Cancer Cell (2012) 22(6):737–50. doi: 10.1016/j.ccr.2012.10.025 PMC3568632 23201164

[43] Storz P. Acinar cell plasticity and development of pancreatic ductal adenocarcinoma, nature reviews. Gastroenterol Hepatol (2017) 14(5):296–304. doi: 10.1038/nrgastro.2017.12 PMC6036907 28270694

[44] Hayakawa Y Sakitani K Konishi M Asfaha S Niikura R Tomita H. Nerve growth factor promotes gastric tumorigenesis through aberrant cholinergic signaling. Cancer Cell (2017) 31(1):21–34. doi: 10.1016/j.ccell.2016.11.005 27989802 PMC5225031

[45] Zhu Z Friess H diMola FF Zimmermann A Graber HU Korc M. Nerve growth factor expression correlates with perineural invasion and pain in human pancreatic cancer. J Clin Oncol (1999) 17(8):2419–28. doi: 10.1200/JCO.1999.17.8.2419 10561305

[46] Sahni S Moon EA Howell VM Mehta S Pavlakis N Chan D. Tissue biomarker panel as a surrogate marker for squamous subtype of pancreatic cancer. Eur J Surg Oncol (2020) 46(8):1539–42. doi: 10.1016/j.ejso.2020.02.001 32061458

[47] Yang A Zylberberg HM Rustgi SD Amin SP Bar-Mashiah A Boffetta P. Beta-blockers have no impact on survival in pancreatic ductal adenocarcinoma prior to cancer diagnosis. Sci Rep (2021) 11(1):1038. doi: 10.1038/s41598-020-80570-0 33441781 PMC7807087

[48] Saad A Goldstein J Margalit O Shacham-Shmueli E Lawrence YR Yang YX. Assessing the effects of beta-blockers on pancreatic cancer risk: A nested case-control study. Pharmacoepidemiology Drug Saf (2020) 29(5):599–604. doi: 10.1002/pds.4993 32196836

[49] Fasanella KE Christianson JA Chanthaphavong RS Davis BM. Distribution and neurochemical identification of pancreatic afferents in the mouse. J Comp Neurol (2008) 509(1):42–52. doi: 10.1002/cne.21736 18418900 PMC2677067

[50] Li Q Peng J. Sensory nerves and pancreatitis. Gland Surg (2014) 3(4):284–92. doi: 10.3978/j.issn.2227-684X.2013.10.08 PMC4244502 25493260

[51] Mehlen P Delloye-Bourgeois C Chédotal A. Novel roles for slits and netrins: axon guidance cues as anticancer targets? Nat Rev Cancer (2011) 11(3):188–97. doi: 10.1038/nrc3005 21326323

[52] Caterina MJ Schumacher MA Tominaga M Rosen TA Levine JD Julius D. The capsaicin receptor: a heat-activated ion channel in the pain pathway. Nature (1997) 389(6653):816–24. doi: 10.1038/39807 9349813

[53] Akiba Y Kato S Katsube K Nakamura M Takeuchi K Ishii H. Transient receptor potential vanilloid subfamily 1 expressed in pancreatic islet beta cells modulates insulin secretion in rats. Biochem Biophys Res Commun (2004) 321(1):219–25. doi: 10.1016/j.bbrc.2004.06.149 15358238

[54] Steinhoff MS von Mentzer B Geppetti P Pothoulakis C Bunnett NW. Tachykinins and their receptors: contributions to physiological control and the mechanisms of disease. Physiol Rev (2014) 94(1):265–301. doi: 10.1152/physrev.00031.2013 24382888 PMC3929113

[55] Singh D Joshi DD Hameed M Qian J Gascón P Maloof PB. Increased expression of preprotachykinin-I and neurokinin receptors in human breast cancer cells: implications for bone marrow metastasis. Proc Natl Acad Sci United States America (2000) 97(1):388–93. doi: 10.1073/pnas.97.1.388 PMC26673 10618428

[56] Garcia-Recio S Fuster G Fernandez-Nogueira P Pastor-Arroyo EM Park SY Mayordomo C. Substance p autocrine signaling contributes to persistent HER2 activation that drives malignant progression and drug resistance in breast cancer. Cancer Res (2013) 73(21):6424–34. doi: 10.1158/0008-5472.CAN-12-4573 24030979

[57] Koon HW Zhao D Zhan Y Rhee SH Moyer MP Pothoulakis C. Substance p stimulates cyclooxygenase-2 and prostaglandin E2 expression through JAK-STAT activation in human colonic epithelial cells. J Immunol (2006) 176(8):5050–9. doi: 10.4049/jimmunol.176.8.5050 PMC2593099 16585602

[58] Pierce KL Luttrell LM Lefkowitz RJ. New mechanisms in heptahelical receptor signaling to mitogen activated protein kinase cascades. Oncogene (2001) 20(13):1532–9. doi: 10.1038/sj.onc.1204184 11313899

[59] Hu CP Feng JT Tang YL Zhu JQ Lin MJ Yu ME. LIF upregulates expression of NK-1R in NHBE cells. Mediators Inflammation (2006) 2006(5):84829. doi: 10.1155/MI/2006/84829 PMC1657075 17392578

[60] Morris HR Panico M Etienne T Tippins J Girgis SI MacIntyre I. Isolation and characterization of human calcitonin gene-related peptide. Nature (1984) 308(5961):746–8. doi: 10.1038/308746a0 6609312

[61] Brain SD Grant AD. Vascular actions of calcitonin gene-related peptide and adrenomedullin. Physiol Rev (2004) 84(3):903–34. doi: 10.1152/physrev.00037.2003 15269340

[62] Nelson MT Huang Y Brayden JE Hescheler J Standen NB. Arterial dilations in response to calcitonin gene-related peptide involve activation of k+ channels. Nature (1990) 344(6268):770–3. doi: 10.1038/344770a0 2109832

[63] Reed WR Little JW Lima CR Sorge RE Yarar-Fisher C Eraslan M. Spinal mobilization prevents NGF-induced trunk mechanical hyperalgesia and attenuates expression of CGRP. Front Neurosci (2020) 14:385. doi: 10.3389/fnins.2020.00385 32425750 PMC7204433

[64] Gao X Zhang D Xu C Li H Caron KM Frenette PS. Nociceptive nerves regulate haematopoietic stem cell mobilization. Nature (2021) 589(7843):591–6. doi: 10.1038/s41586-020-03057-y PMC7856173 33361809

[65] Zhu W Sheng D Shao Y Zhang Q Peng Y. Neuronal calcitonin gene-related peptide promotes prostate tumor growth in the bone microenvironment. Peptides (2021) 135:170423. doi: 10.1016/j.peptides.2020.170423 33086087

[66] Gluexam T Grandits AM Schlerka A Nguyen CH Etzler J Finkes T. CGRP signaling *via* CALCRL increases chemotherapy resistance and stem cell properties in acute myeloid leukemia. Int J Mol Sci (2019) 20(23):5826. doi: 10.3390/ijms20235826 31756985 PMC6928760

[67] Han L Jiang J Xue M Qin T Xiao Y Wu E. Sonic hedgehog signaling pathway promotes pancreatic cancer pain *via* nerve growth factor. Regional Anesth Pain Med (2020) 45(2):137–44. doi: 10.1136/rapm-2019-100991 31792027

[68] Boj SF Hwang CI Baker LA Chio II D.D. Engle Corbo V. Organoid models of human and mouse ductal pancreatic cancer. Cell (2015) 160(1-2):324–38. doi: 10.1016/j.cell.2014.12.021 PMC4334572 25557080

[69] Fischer MJM Ciotu CI Szallasi A. The mysteries of capsaicin-sensitive afferents. Front Physiol (2020) 11:554195. doi: 10.3389/fphys.2020.554195 33391007 PMC7772409

[70] Cabezón-Gutiérrez L Custodio-Cabello S Palka-Kotlowska M Khosravi-Shahi P. High-dose 8% capsaicin patch in treatment of chemotherapy-induced peripheral neuropathy. a systematic review. J Pain symptom Manage (2020) 60(5):1047–1054.e1. doi: 10.1016/j.jpainsymman.2020.06.026 32659321

[71] Schwartz ES La JH Scheff NN Davis BM Albers KM Gebhart GF. TRPV1 and TRPA1 antagonists prevent the transition of acute to chronic inflammation and pain in chronic pancreatitis. J Neurosci (2013) 33(13):5603–11. doi: 10.1523/JNEUROSCI.1806-12.2013 PMC3690366 23536075

[72] Nikolaeva-Koleva M Butron L González-Rodríguez S Devesa I Valente P Serafini M. A capsaicinoid-based soft drug, AG1529, for attenuating TRPV1-mediated histaminergic and inflammatory sensory neuron excitability. Sci Rep (2021) 11(1):246. doi: 10.1038/s41598-020-80725-z 33420359 PMC7794549

[73] Ahrén B. Autonomic regulation of islet hormone secretion–implications for health and disease. Diabetologia (2000) 43(4):393–410. doi: 10.1007/s001250051322 10819232

[74] Lin EE Scott-Solomon E Kuruvilla R. Peripheral innervation in the regulation of glucose homeostasis. Trends Neurosci (2021) 44(3):189–202. doi: 10.1016/j.tins.2020.10.015 33229051 PMC7904596

[75] Rodriguez-Diaz R Abdulreda MH Formoso AL Gans I Ricordi C Berggren PO. Innervation patterns of autonomic axons in the human endocrine pancreas. Cell Metab (2011) 14(1):45–54. doi: 10.1016/j.cmet.2011.05.008 21723503 PMC3135265

[76] Paleari L Grozio A Cesario A Russo P. The cholinergic system and cancer. Semin Cancer Biol (2008) 18(3):211–7. doi: 10.1016/j.semcancer.2007.12.009 18262434

[77] Fredriksson R Lagerström MC Lundin LG Schiöth HB. The G-protein-coupled receptors in the human genome form five main families. phylogenetic analysis, paralogon groups, and fingerprints. Mol Pharmacol (2003) 63(6):1256–72. doi: 10.1124/mol.63.6.1256 12761335

[78] Kruse AC Kobilka BK Gautam D Sexton PM Christopoulos A Wess J. Muscarinic acetylcholine receptors: novel opportunities for drug development, nature reviews. Drug Discovery (2014) 13(7):549–60. doi: 10.1038/nrd4295 PMC5818261 24903776

[79] Zhang L Xiu D Zhan J He X Guo L Wang J. High expression of muscarinic acetylcholine receptor 3 predicts poor prognosis in patients with pancreatic ductal adenocarcinoma. OncoTargets Ther (2016) 9:6719–26. doi: 10.2147/OTT.S111382 PMC5096762 27826198

[80] Park YS Liu Z Vasamsetti BM Cho NJ. The ERK1/2 and mTORC1 signaling pathways are involved in the muscarinic acetylcholine receptor-mediated proliferation of SNU-407 colon cancer cells. J Cell Biochem (2016) 117(12):2854–63. doi: 10.1002/jcb.25597 27167250

[81] Lin G Sun L Wang R Guo Y Xie C. Overexpression of muscarinic receptor 3 promotes metastasis and predicts poor prognosis in non-small-cell lung cancer. J Thorac Oncol (2014) 9(2):170–8. doi: 10.1097/JTO.0000000000000066 PMC4132044 24419413

[82] Hutchings C Phillips JA Djamgoz MBA. Nerve input to tumours: Pathophysiological consequences of a dynamic relationship, biochimica et biophysica acta. Rev Cancer (2020) 1874(2):188411. doi: 10.1124/mol.63.6.1256 32828885

[83] Yang MW Tao LY Jiang YS Yang JY Huo YM Liu DJ. Perineural invasion reprograms the immune microenvironment through cholinergic signaling in pancreatic ductal adenocarcinoma. Cancer Res (2020) 80(10):1991–2003. doi: 10.1158/0008-5472.CAN-19-2689 32098780

[84] Al-Wadei MH Banerjee J Al-Wadei HA Schuller HM. Nicotine induces self-renewal of pancreatic cancer stem cells *via* neurotransmitter-driven activation of sonic hedgehog signalling. Eur J Cancer (2016) 52:188–96. doi: 10.1016/j.ejca.2015.10.003 PMC4698183 26689865

[85] Renz BW Tanaka T Sunagawa M Takahashi R Jiang Z Macchini M. Cholinergic signaling *via* muscarinic receptors directly and indirectly suppresses pancreatic tumorigenesis and cancer stemness. Cancer Discovery (2018) 8(11):1458–73. doi: 10.1158/2159-8290.CD-18-0046 PMC6214763 30185628

[86] Tanaka A Sakaguchi S. Regulatory T cells in cancer immunotherapy. Cell Res (2017) 27(1):109–18. doi: 10.1038/cr.2016.151 PMC5223231 27995907

[87] Chen L Han X. Anti-PD-1/PD-L1 therapy of human cancer: past, present, and future. J Clin Invest (2015) 125(9):3384–91. doi: 10.1172/JCI80011 PMC4588282 26325035

[88] Humpton TJ Alagesan B DeNicola GM Lu D Yordanov GN Leonhardt CS. Oncogenic KRAS induces NIX-mediated mitophagy to promote pancreatic cancer. Cancer Discovery (2019) 9(9):1268–87. doi: 10.1158/2159-8290.CD-18-1409 PMC6726540 31263025

[89] Siska PJ Singer K Evert K Renner K Kreutz M. The immunological warburg effect: Can a metabolic-tumor-stroma score (MeTS) guide cancer immunotherapy? Immunol Rev (2020) 295(1):187–202. doi: 10.1111/imr.12846 32157706

[90] Shiratori R Furuichi K Yamaguchi M Miyazaki N Aoki H Chibana H. Glycolytic suppression dramatically changes the intracellular metabolic profile of multiple cancer cell lines in a mitochondrial metabolism-dependent manner. Sci Rep (2019) 9(1):18699. doi: 10.1038/s41598-019-55296-3 31822748 PMC6904735

[91] Hindson J. Neuronal innervation supports PDAC growth *via* release of serine, nature reviews. Gastroenterol Hepatol (2021) 18(1):5. doi: 10.1038/s41575-020-00394-1 33257832

[92] Perera RM Stoykova S Nicolay BN Ross KN Fitamant J Boukhali M. Transcriptional control of autophagy–lysosome function drives pancreatic cancer metabolism. Nature (2015) 524(7565):361–5. doi: 10.1038/nature14587 PMC5086585 26168401

[93] Hong X Zhong L Xie Y Zheng K Pang J Li Y. Matrine reverses the warburg effect and suppresses colon cancer cell growth *via* negatively regulating HIF-1α. Front Pharmacol (2019) 10:1437. doi: 10.3389/fphar.2019.01437 31849679 PMC6892950

[94] Andersen JL Kornbluth S. The tangled circuitry of metabolism and apoptosis. Mol Cell (2013) 49(3):399–410. doi: 10.1016/j.molcel.2012.12.026 23395270 PMC3801185

[95] Henneberger C Papouin T Oliet SH Rusakov DA. Long-term potentiation depends on release of d-serine from astrocytes. Nature (2010) 463(7278):232–6. doi: 10.1038/nature08673 PMC2807667 20075918

[96] Van Horn MR Strasser A Miraucourt LS Pollegioni L Ruthazer ES. The gliotransmitter d-serine promotes synapse maturation and axonal stabilization *In vivo* . J Neurosci (2017) 37(26):6277–88. doi: 10.1523/JNEUROSCI.3158-16.2017 PMC6705694 28550169

[97] Satriano L Lewinska M Rodrigues PM Banales JM Andersen JB. Metabolic rearrangements in primary liver cancers: cause and consequences, nature reviews. Gastroenterol Hepatol (2019) 16(12):748–66. doi: 10.1038/s41575-019-0217-8 31666728

[98] Bergers G Fendt SM. The metabolism of cancer cells during metastasis, nature reviews. Cancer (2021) 21(3):162–80. doi: 10.1038/s41568-020-00320-2 PMC8733955 33462499

[99] Tadros S Shukla SK King RJ Gunda V Vernucci E Abrego J. *De novo* lipid synthesis facilitates gemcitabine resistance through endoplasmic reticulum stress in pancreatic cancer. Cancer Res (2017) 77(20):5503–17. doi: 10.1158/0008-5472.CAN-16-3062 PMC5645242 28811332

[100] Abrego J Gunda V Vernucci E Shukla SK King RJ Dasgupta A. GOT1-mediated anaplerotic glutamine metabolism regulates chronic acidosis stress in pancreatic cancer cells. Cancer Lett (2017) 400:37–46. doi: 10.1016/j.canlet.2017.04.029 28455244 PMC5488721

[101] Fox RG Lytle NK Jaquish DV Park FD Ito T Bajaj J. Image-based detection and targeting of therapy resistance in pancreatic adenocarcinoma. Nature (2016) 534(7607):407–11. doi: 10.1038/nature17988 PMC4998062 27281208

[102] Warburg O. [Origin of cancer cells]. Oncologia (1956) 9(2):75–83. doi: 10.1159/000223920 13335077

[103] Ying H Kimmelman AC Lyssiotis CA Hua S Chu GC Fletcher-Sananikone E. Oncogenic kras maintains pancreatic tumors through regulation of anabolic glucose metabolism. Cell (2012) 149(3):656–70. doi: 10.1016/j.cell.2012.01.058 PMC3472002 22541435

[104] Achalandabaso Boira M Di Martino M Gordillo C Adrados M Martín-Pérez E. GLUT-1 as a predictor of worse prognosis in pancreatic adenocarcinoma: immunohistochemistry study showing the correlation between expression and survival. BMC Cancer (2020) 20(1):909. doi: 10.21203/rs.3.rs-18270/v1 32967636 PMC7510075

[105] Ding L Madamsetty VS Kiers S Alekhina O Ugolkov A Dube J. Glycogen synthase kinase-3 inhibition sensitizes pancreatic cancer cells to chemotherapy by abrogating the TopBP1/ATR-mediated DNA damage response. Clin Cancer Res (2019) 25(21):6452–62. doi: 10.1158/1078-0432.CCR-19-0799 PMC6825568 31533931

[106] Yang X Qian K. Protein O-GlcNAcylation: emerging mechanisms and functions, nature reviews. Mol Cell Biol (2017) 18(7):452–65. doi: 10.1038/nrm.2017.22 PMC5667541 28488703

[107] Sharma NS Gupta VK Garrido VT Hadad R Durden BC Kesh K. Targeting tumor-intrinsic hexosamine biosynthesis sensitizes pancreatic cancer to anti-PD1 therapy. J Clin Invest (2020) 130(1):451–65. doi: 10.1172/JCI127515 PMC6934212 31613799

[108] Yang M Vousden KH. Serine and one-carbon metabolism in cancer, nature reviews. Cancer (2016) 16(10):650–62. doi: 10.1038/nrc.2016.81 27634448

[109] Mauro VP Chappell SA. A critical analysis of codon optimization in human therapeutics. Trends Mol Med (2014) 20(11):604–13. doi: 10.1016/j.molmed.2014.09.003 PMC4253638 25263172

[110] Ma X Li B Liu J Fu Y Luo Y. Phosphoglycerate dehydrogenase promotes pancreatic cancer development by interacting with eIF4A1 and eIF4E. J Exp Clin Cancer Res (2019) 38(1):66. doi: 10.1186/s13046-019-1053-y 30744688 PMC6371491

[111] Zhou YY Chen LP Zhang Y Hu SK Dong ZJ Wu M. Integrated transcriptomic analysis reveals hub genes involved in diagnosis and prognosis of pancreatic cancer. Mol Med (2019) 25(1):47. doi: 10.1186/s10020-019-0113-2 31706267 PMC6842480

[112] Ross KC Andrews AJ Marion CD Yen TJ Bhattacharjee V. Identification of the serine biosynthesis pathway as a critical component of BRAF inhibitor resistance of melanoma, pancreatic, and non-small cell lung cancer cells. Mol Cancer Ther (2017) 16(8):1596–609. doi: 10.1158/1535-7163.MCT-16-0798 PMC5544579 28500236

[113] Sullivan MR Mattaini KR Dennstedt EA Nguyen AA Sivanand S Reilly MF. Increased serine synthesis provides an advantage for tumors arising in tissues where serine levels are limiting. Cell Metab (2019) 29(6):1410–1421.e4. doi: 10.1016/j.cmet.2019.02.015 30905671 PMC6551255

[114] Bialopiotrowicz E Noyszewska-Kania M Kachamakova-Trojanowska N Loboda A Cybulska M Grochowska A. Serine biosynthesis pathway supports MYC-miR-494-EZH2 feed-forward circuit necessary to maintain metabolic and epigenetic reprogramming of burkitt lymphoma cells. Cancers (2020) 12(3):580. doi: 10.3390/cancers12030580 32138178 PMC7139810

[115] Song Z Feng C Lu Y Lin Y Dong C. PHGDH is an independent prognosis marker and contributes cell proliferation, migration and invasion in human pancreatic cancer. Gene (2018) 642:43–50. doi: 10.1016/j.gene.2017.11.014 29128633

[116] Metcalf S Dougherty S Kruer T Hasan N Biyik-Sit R Reynolds L. Selective loss of phosphoserine aminotransferase 1 (PSAT1) suppresses migration, invasion, and experimental metastasis in triple negative breast cancer. Clin Experiment Metastasis (2020) 37(1):187–97. doi: 10.1007/s10585-019-10000-7 31630284

[117] Rawat V Malvi P Della Manna D Yang ES Bugide S Zhang X. PSPH promotes melanoma growth and metastasis by metabolic deregulation-mediated transcriptional activation of NR4A1. Oncogene (2021) 40(13):2448–62. doi: 10.1038/s41388-021-01683-y PMC8026604 33674745

[118] Dekhne AS Ning C Nayeen MJ Shah K Kalpage H Frühauf J. Cellular pharmacodynamics of a novel Pyrrolo[3,2-d]pyrimidine inhibitor targeting mitochondrial and cytosolic one-carbon metabolism. Mol Pharmacol (2020) 97(1):9–22. doi: 10.1124/mol.119.117937 31707355 PMC6877291

[119] Dekhne AS Shah K Ducker GS Katinas JM Wong-Roushar J Nayeen MJ. Novel Pyrrolo[3,2-d]pyrimidine compounds target mitochondrial and cytosolic one-carbon metabolism with broad-spectrum antitumor efficacy. Mol Cancer Ther (2019) 18(10):1787–99. doi: 10.1158/1535-7163.MCT-19-0037 PMC6774887 31289137

[120] Wang JQ Wang LY Li SJ Tong T Wang L Huang CS. Histone methyltransferase G9a inhibitor-loaded redox-responsive nanoparticles for pancreatic ductal adenocarcinoma therapy. Nanoscale (2020) 12(29):15767–74. doi: 10.1039/D0NR03138K 32729861

[121] Sun HW Yu XJ Wu WC Chen J Shi M Zheng L. GLUT1 and ASCT2 as predictors for prognosis of hepatocellular carcinoma. PloS One (2016) 11(12):e0168907. doi: 10.1371/journal.pone.0168907 28036362 PMC5201247

[122] Sharma A Smyrk TC Levy MJ Topazian MA Chari ST. Fasting blood glucose levels provide estimate of duration and progression of pancreatic cancer before diagnosis. Gastroenterology (2018) 155(2):490–500.e2. doi: 10.1053/j.gastro.2018.04.025 29723506 PMC6067966

[123] Yan L Raj P Yao W Ying H. Glucose metabolism in pancreatic cancer. J Biol Chem (2003) 11(10). 10.3390/cancers11101460 PMC6826406 31569510

[124] Penheiter AR Deelchand DK Kittelson E Damgard SE Murphy SJ O'Brien DR. Identification of a pyruvate-to-lactate signature in pancreatic intraductal papillary mucinous neoplasms. Pancreatology (2018) 18(1):46–53. doi: 10.1016/j.pan.2017.11.006 29170050 PMC6139027

[125] McCommis KS Chen Z Fu X McDonald WG Colca JR Kletzien RF. Loss of mitochondrial pyruvate carrier 2 in the liver leads to defects in gluconeogenesis and compensation *via* pyruvate-alanine cycling. Cell Metab (2015) 22(4):682–94. doi: 10.1016/j.cmet.2015.07.028 PMC4598280 26344101

[126] Cortese N Capretti G Barbagallo M Rigamonti A Takis PG Castino GF. Metabolome of pancreatic juice delineates distinct clinical profiles of pancreatic cancer and reveals a link between glucose metabolism and PD-1(+) cells. Cancer Immunol Res (2020) 8(4):493–505. doi: 10.1158/2326-6066.CIR-19-0403 32019781

[127] Bhagat TD Von Ahrens D Dawlaty M Zou Y Baddour J Achreja A. Lactate-mediated epigenetic reprogramming regulates formation of human pancreatic cancer-associated fibroblasts. eLife (2019) 8:e50663. doi: 10.7554/eLife.50663 31663852 PMC6874475

[128] Brockmueller A Sameri S Liskova A Zhai K Varghese E Samuel SM. Resveratrol's anti-cancer effects through the modulation of tumor glucose metabolism. Cancers (2021) 13(2):188. doi: 10.3390/cancers13020188 33430318 PMC7825813

[129] Anderson M Marayati R Moffitt R Yeh JJ. Hexokinase 2 promotes tumor growth and metastasis by regulating lactate production in pancreatic cancer. Oncotarget (2017) 8(34):56081–94. doi: 10.18632/oncotarget.9760 PMC5593546 28915575

[130] Amendola CR Mahaffey JP Parker SJ Ahearn IM Chen WC Zhou M. KRAS4A directly regulates hexokinase 1. Nature (2019) 576(7787):482–6. doi: 10.1038/s41586-019-1832-9 PMC6923592 31827279

[131] Tsutsumi S Yanagawa T Shimura T Kuwano H Raz A. Autocrine motility factor signaling enhances pancreatic cancer metastasis. Clin Cancer Res (2004) 10(22):7775–84. doi: 10.1158/1078-0432.CCR-04-1015 15570012

[132] Badea L Herlea V Dima SO Dumitrascu T Popescu I. Combined gene expression analysis of whole-tissue and microdissected pancreatic ductal adenocarcinoma identifies genes specifically overexpressed in tumor epithelia. Hepato-gastroenterology (2008) 55(88):2016–27. 19260470

[133] Moir JAG Long A Haugk B French JJ Charnley RM Manas DM. Therapeutic strategies toward lactate dehydrogenase within the tumor microenvironment of pancreatic cancer. Pancreas (2020) 49(10):1364–71. doi: 10.1097/MPA.0000000000001689 33122526

[134] Maftouh M Avan A Sciarrillo R Granchi C Leon LG Rani R. Synergistic interaction of novel lactate dehydrogenase inhibitors with gemcitabine against pancreatic cancer cells in hypoxia. Br J Cancer (2014) 110(1):172–82. doi: 10.1038/bjc.2013.681 PMC3887288 24178759

[135] Ponsuksili S Trakooljul N Hadlich F Methling K Lalk M Murani E. Genetic regulation of liver metabolites and transcripts linking to biochemical-clinical parameters. Front Genet (2019) 10:348. doi: 10.3389/fgene.2019.00348 31057604 PMC6478805

[136] Schrader H Menge BA Belyaev O Uhl W Schmidt WE Meier JJ. Amino acid malnutrition in patients with chronic pancreatitis and pancreatic carcinoma. Pancreas (2009) 38(4):416–21. doi: 10.1097/MPA.0b013e318194fc7a 19169171

[137] Parker SJ Amendola CR Hollinshead KER Yu Q Yamamoto K Encarnacion-Rosado J. Selective alanine transporter utilization creates a targetable metabolic niche in pancreatic cancer. Cancer Discovery (2020) 10(7):1018–37. doi: 10.1158/2159-8290.CD-19-0959 PMC7334074 32341021

[138] Dong S Wang L Guo YB Ying HF Shen XH Meng ZQ. Risk factors of liver metastasis from advanced pancreatic adenocarcinoma: a large multicenter cohort study. World J Surg Oncol (2017) 15(1):120. doi: 10.1186/s12957-016-1064-5 28673297 PMC5496221

[139] Riedl JM Posch F Prager G Eisterer W Oehler L Sliwa T. The AST/ALT (De ritis) ratio predicts clinical outcome in patients with pancreatic cancer treated with first-line nab-paclitaxel and gemcitabine: *post hoc* analysis of an Austrian multicenter, noninterventional study. Ther Adv Med Oncol (2020) 12:1758835919900872. doi: 10.1177/1758835919900872 32313566 PMC7153180

[140] Fu Y Ding L Yang X Ding Z Huang X Zhang L. Asparagine synthetase-mediated l-asparagine metabolism disorder promotes the perineural invasion of oral squamous cell carcinoma. Front Oncol (2021) 11:637226. doi: 10.3389/fonc.2021.637226 33777794 PMC7987891

[141] Zheng M Guo J Xu J Yang K Tang R Gu X. Ixocarpalactone a from dietary tomatillo inhibits pancreatic cancer growth by targeting PHGDH. Food Funct (2019) 10(6):3386–95. doi: 10.1039/C9FO00394K 31112178

[142] Yan Y Huang P Mao K He C Xu Q Zhang M. Anti-oncogene PTPN13 inactivation by hepatitis b virus X protein counteracts IGF2BP1 to promote hepatocellular carcinoma progression. Oncogene (2021) 40(1):28–45. doi: 10.1038/s41388-020-01498-3 33051595 PMC7790756

[143] Ceyhan GO Bergmann F Kadihasanoglu M Altintas B Demir IE Hinz U. Pancreatic neuropathy and neuropathic pain–a comprehensive pathomorphological study of 546 cases. Gastroenterology (2009) 136(1):177–186.e1. doi: 10.1053/j.gastro.2008.09.029 18992743

[144] Hessmann E Buchholz SM Demir IE Singh SK Gress TM Ellenrieder V. Microenvironmental determinants of pancreatic cancer. Physiol Rev (2020) 100(4):1707–51. doi: 10.1152/physrev.00042.2019 32297835

[145] Demir IE Friess H Ceyhan GO. Nerve-cancer interactions in the stromal biology of pancreatic cancer. Front Physiol (2012) 3:97. doi: 10.3389/fphys.2012.00097 22529816 PMC3327893

[146] Jiang SH Li J Dong FY Yang JY Liu DJ Yang XM. Increased serotonin signaling contributes to the warburg effect in pancreatic tumor cells under metabolic stress and promotes growth of pancreatic tumors in mice. Gastroenterology (2017) 153(1):277–291.e19. doi: 10.1053/j.gastro.2017.03.008 28315323

[147] Sousa CM Biancur DE Wang X Halbrook CJ Sherman MH Zhang L. Pancreatic stellate cells support tumour metabolism through autophagic alanine secretion. Nature (2016) 536(7617):479–83. doi: 10.1038/nature19084 PMC5228623 27509858

[148] Taylor AM Blurton-Jones M Rhee SW Cribbs DH Cotman CW Jeon NL. A microfluidic culture platform for CNS axonal injury, regeneration and transport. Nat Methods (2005) 2(8):599–605. doi: 10.1038/nmeth777 16094385 PMC1558906

[149] Harjes U. The neuronal-metabolic interface, nature reviews. Cancer (2021) 21(2):68. doi: 10.1038/s41568-020-00324-y 33262455

[150] Schneider-Poetsch T Ju J Eyler DE Dang Y Bhat S Merrick WC. Inhibition of eukaryotic translation elongation by cycloheximide and lactimidomycin. Nat Chem Biol (2010) 6(3):209–17. doi: 10.1038/nchembio.304 PMC2831214 20118940

[151] Li X Zhao X Fang Y Jiang X Duong T Fan C. Generation of destabilized green fluorescent protein as a transcription reporter. J Biol Chem (1998) 273(52):34970–5. doi: 10.1074/jbc.273.52.34970 9857028

[152] Ingolia NT Brar GA Rouskin S McGeachy AM Weissman JS. The ribosome profiling strategy for monitoring translation *in vivo* by deep sequencing of ribosome-protected mRNA fragments. Nat Protoc (2012) 7(8):1534–50. doi: 10.1038/nprot.2012.086 PMC3535016 22836135

[153] Ghilardi JR Freeman KT Jimenez-Andrade JM Mantyh WG Bloom AP Kuskowski MA. Administration of a tropomyosin receptor kinase inhibitor attenuates sarcoma-induced nerve sprouting, neuroma formation and bone cancer pain. Mol Pain (2010) 6:87. doi: 10.1186/1744-8069-6-87 21138586 PMC3004846

[154] Gu I Gregory E Atwood C Lee SO Song YH. Exploring the role of metabolites in cancer and the associated nerve crosstalk. Nutrients (2022) 14(9):1722. doi: 10.3390/nu14091722 35565690 PMC9103817

[155] Nusser N Gosmanova E Makarova N Fujiwara Y Yang L Guo F. Serine phosphorylation differentially affects RhoA binding to effectors: implications to NGF-induced neurite outgrowth. Cell signalling (2006) 18(5):704–14. doi: 10.1016/j.cellsig.2005.06.010 16109481

[156] Zhou L Too HP. Mitochondrial localized STAT3 is involved in NGF induced neurite outgrowth. PloS One (2011) 6(6):e21680. doi: 10.1371/journal.pone.0021680 21738764 PMC3124549

[157] Kim E Jung S Park WS Lee JH Shin R Heo SC. Upregulation of SLC2A3 gene and prognosis in colorectal carcinoma: analysis of TCGA data. BMC Cancer (2019) 19(1):302. doi: 10.1186/s12885-019-5475-x 30943948 PMC6446261

[158] Liu J Hong J Han H Park J Kim D Park H. Decreased vitamin c uptake mediated by SLC2A3 promotes leukaemia progression and impedes TET2 restoration. Br J Cancer (2020) 122(10):1445–52. doi: 10.1038/s41416-020-0788-8 PMC7217885 32203209

